# Clinical manifestations and proportions of severe outcomes in pediatric acute post-streptococcal glomerulonephritis: a systematic review and meta-analysis of predominantly hospital-based cohorts

**DOI:** 10.3389/fmed.2026.1953518

**Published:** 2026-09-25

**Authors:** Ronglang Wu, Peng Ren, Zhenglin Chen, Xuefen Kang, Qi Zhang

**Affiliations:** 1 The Affiliated People’s Hospital of Fujian University of Traditional Chinese Medicine, Fuzhou, China; 2 Fujian University of Traditional Chinese Medicine, Fuzhou, China

**Keywords:** acute kidney injury, acute post-streptococcal glomerulonephritis, children, hypertension, meta-analysis, renal replacement therapy

## Abstract

**Objective:**

To systematically evaluate the proportions of major clinical manifestations and severe outcomes in children with acute post-streptococcal glomerulonephritis (APSGN).

**Methods:**

PubMed, Embase, Web of Science, the Cochrane Library, Scopus, and ClinicalTrials.gov were systematically searched for observational studies reporting clinical manifestations or outcomes of APSGN in children. Outcomes of interest included hypertension, gross hematuria, study-defined AKI/acute kidney dysfunction, nephrotic-range proteinuria, severe complications, an exploratory RPGN/crescentic-lesion composite (rapidly progressive glomerulonephritis and/or crescentic lesions), and renal replacement therapy (RRT). Random-effects single-proportion meta-analyses were performed to estimate pooled proportions and 95% confidence intervals (CIs). Robustness was assessed using heterogeneity and sensitivity analyses, with funnel plots and Egger's regression used to evaluate possible small-study effects.

**Results:**

Eighteen studies involving 2,191 children were included, predominantly from APSGN cohorts, with some studies using broader acute glomerulonephritis (AGN) or postinfectious glomerulonephritis (PIGN) definitions. Across these predominantly hospital-based cohorts, hypertension was the most commonly reported clinical manifestation, with a pooled proportion of 65.0% (17 studies, 2,164 children; 95% CI, 53.3%–79.2%). The pooled proportions of gross hematuria, study-defined AKI/acute kidney dysfunction, and nephrotic-range proteinuria were 47.5% (14 studies, 1,665 children; 95% CI, 34.0%–66.4%), 40.1% (18 studies, 2,173 children; 95% CI, 28.4%–56.7%), and 35.9% (11 studies, 887 children; 95% CI, 26.2%–49.0%), respectively. The pooled proportions of severe complications, the exploratory RPGN/crescentic-lesion composite, and RRT were 12.9% (16 studies, 1,722 children; 95% CI, 8.2%–20.3%), 12.9% (10 studies, 699 evaluable children; 95% CI, 4.1%–40.8%), and 4.0% (15 studies, 1,465 children; 95% CI, 1.4%–11.2%), respectively. The exploratory composite combined three clinically defined RPGN entries with seven crescentic-lesion entries and should not be interpreted as the prevalence of either clinical RPGN or crescentic glomerulonephritis separately. Significant between-study heterogeneity was observed for all outcomes (I^2^, 82.2%–97.7%). Sensitivity analyses generally supported the pooled estimates for common manifestations, whereas the RRT estimate and the exploratory RPGN/crescentic-lesion composite estimate were more sensitive to cohort selection. Funnel-plot asymmetry was observed across the evaluated outcomes and was interpreted cautiously as possible small-study effects rather than definitive publication bias.

**Conclusion:**

Hypertension, gross hematuria, study-defined AKI/acute kidney dysfunction, and nephrotic-range proteinuria were commonly reported across the predominantly hospital-based cohorts, whereas RRT and severe complications were reported less often. The heterogeneous exploratory RPGN/crescentic-lesion composite should not be interpreted as the prevalence of either clinical RPGN or crescentic glomerulonephritis separately. Given the clinically heterogeneous evidence base and substantial statistical uncertainty, these pooled proportions summarize published clinical cohorts and are not stable population-level frequencies applicable to all children with APSGN. Close monitoring of blood pressure, volume status, and kidney function is warranted during the acute phase.

**Systematic Review Registration:**

https://www.crd.york.ac.uk/PROSPERO/view/CRD420261464457. PROSPERO CRD420261464457.

## Introduction

Acute post-streptococcal glomerulonephritis (APSGN) is an important cause of acute glomerulonephritis in children (1–3). Previous global burden estimates suggested that approximately 472,000 cases of APSGN and 5,000 associated deaths occur worldwide each year, with the vast majority of cases arising in resource-limited settings (4). The annual incidence of APSGN among children is approximately 24.3 per 100,000 in resource-limited settings, substantially higher than the approximately 6.2 per 100,000 reported in resource-rich settings (3, 4). With improvements in sanitation, access to clean water, and prevention and control of streptococcal infections, the incidence of APSGN has gradually declined in high-income countries. Nevertheless, the disease continues to impose a considerable burden in low- and middle-income countries and among certain Indigenous and socioeconomically disadvantaged populations (3, 5, 6).

The clinical presentation and severity of APSGN vary widely. Affected children may present with hematuria, edema, hypertension, proteinuria, and varying degrees of kidney dysfunction; some may also develop acute kidney injury (AKI), nephrotic-range proteinuria, rapidly progressive glomerulonephritis (RPGN), or crescentic lesions (7–9). Volume overload and severe hypertension may result in hypertensive encephalopathy, pulmonary edema, and heart failure, and a small proportion of critically ill children require renal replacement therapy (RRT) (9–11). Although most children recover gradually with supportive care, severe kidney injury and acute cardiopulmonary or neurologic complications may substantially increase the burden of disease (9–11).

Previous pediatric observational studies have described the clinical characteristics and outcomes of APSGN; however, the reported proportions of hypertension, gross hematuria, AKI, nephrotic-range proteinuria, RRT, and severe complications vary considerably across studies (12, 13). These differences may reflect variation in geographic and ethnic backgrounds, case sources, hospitalization or referral criteria, disease severity, outcome definitions, and follow-up duration (5, 12, 13). Moreover, most available studies are single-center observational investigations with limited sample sizes, incomplete outcome reporting, and inconsistent methods. Previous reviews have primarily provided narrative summaries of APSGN epidemiology, clinical manifestations, diagnosis, treatment, and prognosis (1–3, 7–9). A systematic quantitative synthesis of the proportions of major clinical manifestations and severe outcomes in children with APSGN remains lacking.

Therefore, this study aimed to provide a descriptive quantitative synthesis of the study-reported proportions of major clinical manifestations and severe outcomes in pediatric APSGN and to assess the robustness and interpretability of these estimates through risk-of-bias assessment and sensitivity analyses.

## Methods

### Study design

This meta-analysis was conducted in accordance with the Preferred Reporting Items for Systematic Reviews and Meta-Analyses (PRISMA) 2020 statement (14). The study protocol was prospectively registered with PROSPERO (registration number: CRD420261464457).

### Search strategy

PubMed, Embase, Web of Science, the Cochrane Library, and Scopus were systematically searched from database inception to July 15, 2026, and ClinicalTrials.gov was searched for relevant registered studies. Database-specific search strategies were developed using disease-specific terminology and combinations of terms related to streptococcal infection and glomerulonephritis, with syntax and search fields adapted to each database. No language restrictions were applied. Reference lists of all included studies were manually screened for additional potentially eligible reports. Complete line-by-line search strategies for each source, including field specifications, Boolean operators, and the number of records retrieved, are provided in the Supplementary Material.

### Eligibility criteria

Studies were eligible if they met all of the following criteria: (1) the population comprised children with APSGN or an identifiable pediatric poststreptococcal/postinfectious glomerulonephritis population within a broader AGN/PIGN cohort; (2) at least one of the following outcomes was reported: AKI, hypertension, gross hematuria, nephrotic-range proteinuria, RRT, clinically defined RPGN, crescentic lesions on kidney biopsy, or other severe complications; (3) the event count and evaluable denominator were extractable; and (4) the study had a prospective or retrospective cohort design. Because diagnostic criteria varied across settings and periods, evidence of preceding streptococcal infection was not retrospectively imposed as a uniform requirement beyond each report's case definition. For each study, we recorded whether such evidence was explicitly required without an alternative diagnostic route; included in the stated criteria but not documented for all participants; part of a broader or alternative clinical, pathological, or laboratory pathway; or insufficiently described to determine a uniform algorithm. These categories were used to describe study-level diagnostic ascertainment rather than to retrospectively reclassify the diagnoses assigned by the original investigators. We also recorded the reported evidence [e.g., antistreptolysin O (ASO), anti-DNase B, throat or skin culture/swab, or compatible clinical or epidemiological history], complement findings, and kidney-biopsy evidence where available. Broader AGN/PIGN cohorts were retained only when the authors identified such a relevant pediatric population and outcome-specific data were extractable. Study-level diagnostic ascertainment is documented in Supplementary Table S1, and the influence of broader or less-specific definitions was examined in a diagnostic-specificity sensitivity analysis.

The exclusion criteria were as follows: (1) non-original publications, including case reports, conference abstracts, theses or dissertations, letters, commentaries, reviews, meta-analyses, editorials, and study protocols; (2) studies that did not include children with APSGN or from which pediatric APSGN data could not be extracted separately; (3) studies with designs that did not meet the inclusion criteria; (4) studies from which the event count and corresponding evaluable sample size could not be extracted; and (5) duplicate publications or studies with clearly overlapping populations.

### Study selection

All retrieved records were imported into EndNote 20 (Clarivate Analytics), and duplicates were removed. Before formal title and abstract screening, records with publication types that clearly did not meet the prespecified eligibility criteria were manually excluded in EndNote; no automated screening tool was used. For this preliminary step, two reviewers independently screened the records and subsequently cross-checked their screening decisions, resulting in the exclusion of 1,095 records. Two reviewers then independently screened the titles and abstracts of the remaining records and assessed the full texts of potentially eligible reports according to the eligibility criteria. Reference lists of included studies were also checked manually for additional eligible reports. When relevant information was not available in the main article, corresponding supplementary materials or registry records were examined. Disagreements were resolved through discussion, with adjudication by a third reviewer when necessary.

### Data extraction

Two reviewers independently extracted data using a prespecified standardized form. The extracted information included: (1) study characteristics, including first author, publication year, country or region, study design, and sample size; (2) participant and setting characteristics, including age, sex distribution, population characteristics, clinical setting, preceding infection, the role of preceding streptococcal evidence in the reported diagnostic pathway and the specific evidence used, complement findings, and follow-up duration; and (3) clinical outcomes, including the event count and evaluable sample size for AKI or acute kidney dysfunction, hypertension, gross hematuria, nephrotic-range proteinuria, RRT, RPGN, crescentic lesions, and severe complications. Any disagreements were resolved through discussion or consultation with a third reviewer. Study investigators were not contacted because the aggregate event counts and corresponding evaluable denominators required for the analyses were obtainable from the published reports, supplementary materials, or registry records.

### Study quality assessment

Two reviewers independently assessed the methodological quality of the included studies using an adapted Newcastle-Ottawa Scale (NOS) for observational cohort studies (15). The scale evaluated study selection, comparability, and outcome assessment, with a maximum score of nine stars. Studies scoring 7–9, 5–6, and 0–4 stars were classified as high, moderate, and low quality, respectively. Disagreements were resolved through discussion or adjudication by a third reviewer. Although prespecified in the registered protocol, ROBINS-I was not used as the primary instrument because it was developed for non-randomized intervention studies; its assessment is retained in Supplementary Table S9 for protocol transparency. The adapted NOS was used for the primary quality assessment of the included observational cohorts.

### Statistical analysis

Statistical analyses were performed in R version 4.4.3 using the meta (version 8.5-0) and metafor (version 5.0-1) packages. A separate single-proportion meta-analysis was conducted for each clinical outcome. Studies that did not report the outcome of interest or lacked the corresponding evaluable sample size were excluded from the analysis of that outcome. Event proportions were natural log-transformed (PLN) and pooled using an inverse-variance random-effects model. A continuity correction of 0.5 was applied only to studies with a boundary proportion (zero events or zero non-events), and individual-study confidence intervals were calculated using the exact Clopper-Pearson method. Between-study variance (tau^2^) was estimated using the DerSimonian-Laird method, and 95% CIs for the pooled estimates were calculated using the Hartung-Knapp adjustment. The DerSimonian-Laird estimator was retained for the primary analysis to preserve consistency with the original analytical framework; model dependence was additionally evaluated using binomial-logit GLMMs.

Between-study heterogeneity was assessed using Cochran's *Q* test, the I^2^ statistic, and tau^2^. Robustness was evaluated using sensitivity analyses addressing cohort selection, diagnostic specificity, AKI definition, study quality, and leave-one-out exclusion. Random-intercept binomial-logit GLMMs were additionally fitted as alternative models, with bounded 95% prediction intervals reported. Prediction intervals displayed in the primary forest plots were derived from the primary inverse-variance random-effects model, whereas Supplementary Table S5 reports bounded GLMM-based prediction intervals as an alternative-model sensitivity analysis. For the exploratory RPGN/crescentic-lesion forest plots, prediction limits exceeding the admissible proportion scale were capped at 100% for display; this graphical cap does not make the underlying PLN model bounded. For outcomes reported by at least 10 studies, funnel plots and Egger's regression tests were used only as exploratory assessments of funnel-plot asymmetry and possible small-study effects. Because these methods have limited reliability when few studies are available and asymmetry may arise from heterogeneity or other mechanisms, they were not interpreted as demonstrating publication bias. All statistical tests were two-sided, and *P* < 0.05 was considered statistically significant.

## Results

### Study selection

The database and clinical trial registry searches identified 2,407 records: 544 from PubMed, 378 from Web of Science, 756 from Embase, 13 from the Cochrane Library, 714 from Scopus, and 2 from ClinicalTrials.gov. After 566 duplicate records were removed, 1,841 records remained for screening.

Before formal title/abstract screening, 1,095 records with publication types that clearly did not meet the prespecified eligibility criteria were manually removed in EndNote, after independent screening by two reviewers followed by cross-checking of their screening decisions; no automated screening tool was used. The titles and abstracts of the remaining 746 records were then assessed, and 723 records that did not meet the eligibility criteria were excluded. Twenty-three reports underwent full-text assessment. Five reports were subsequently excluded: three because of duplicate publication or overlapping study populations, one because adult participants were included, and one because valid data could not be extracted. Ultimately, 18 studies were included, all of which contributed to the quantitative synthesis. The study selection process is shown in Figure 1.

**Figure 1:**
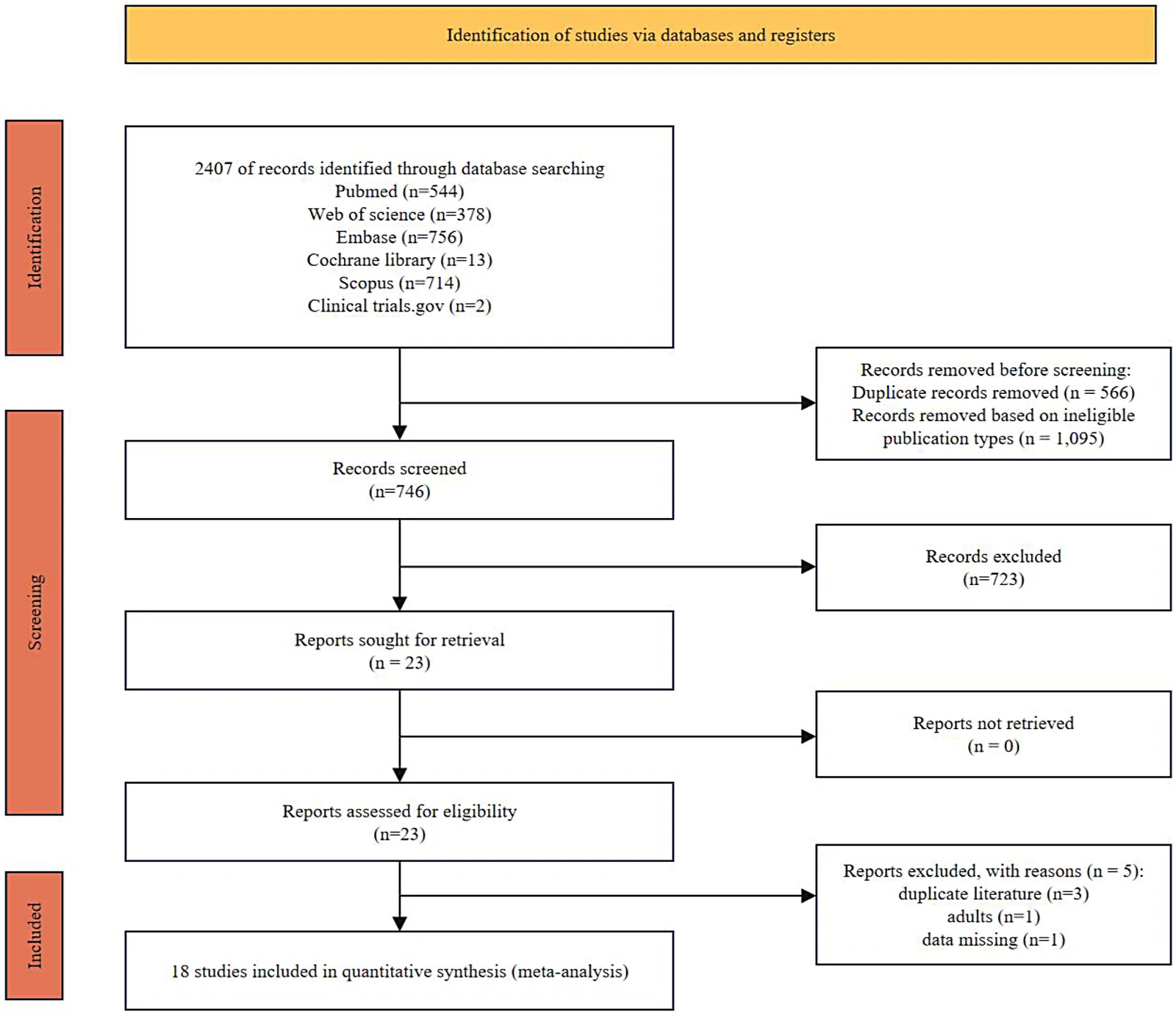
PRISMA flow diagram of study selection.

### Characteristics of the included studies

The 18 included studies involved 2,191 children, predominantly from APSGN cohorts, with some studies using broader acute glomerulonephritis (AGN) or postinfectious glomerulonephritis (PIGN) definitions. Study-level diagnostic ascertainment varied. Seven studies explicitly required evidence of preceding streptococcal infection without a reported alternative diagnostic route. In the remaining studies, streptococcal evidence was incorporated with varying degrees of completeness or could be supplemented by compatible clinical, laboratory, or pathological evidence. These distinctions describe the diagnostic pathways reported in the primary studies rather than a retrospective reclassification of individual cases. Reported evidence included ASO/ASLO, anti-DNase B, throat or skin culture/swab, and compatible clinical or epidemiologic history; complement findings and biopsy evidence were variably used as supportive or alternative criteria. Three cohorts used broader AGN/PIGN definitions. Detailed study-level criteria are provided in Supplementary Table S1, and the influence of broader/less-specific definitions was examined in a diagnostic-specificity sensitivity analysis. The studies were published between 2009 and 2026 and were conducted across multiple countries and regions in Oceania, Asia, Africa, the Middle East, Europe, and North America. Most were retrospective cohort studies: 16 were retrospective, one was prospective, and one included both retrospective and prospective components. Individual study sample sizes ranged from 17 to 400 participants. Reported measures of central tendency for age ranged from 5.0 to 13.0 years, and the proportion of male participants ranged from 51.0% to 74.4%. The evidence base was predominantly hospital-based, including tertiary referral populations and some biopsy-selected or severe-disease-enriched cohorts. Follow-up ranged from the hospitalization period to 5 years. Detailed baseline characteristics are presented in Table 1.

**Table 1:** Baseline characteristics of included studies (*N* = 18 studies, *n* = 2,191 children).

Study (year)	Country/region	Study design	Sample size (*N*)	Age (years)^a^	Male (%)	Population/setting characteristics	Preceding infection (skin/respiratory tract)	Follow-up duration
Wong (2009) (25)	New Zealand	R	27	9.4 (range, 3.5–13.2)	59.3	Māori/Pacific Islander, 85%	NR	Median, 2 years
Becquet (2010) (26)	French Polynesia	R	50	6.7 ± 2.9	54.0	Polynesian, 98%	20.0%/30.0%	Mean, 5 months
Luo (2010)^b^ (27)	China	R	60	13 (median)	58.3	Han Chinese	6.7%/83.3%	6–12 months
Gunasekaran (2015) (13)	India	P	65	6.8 ± 2.8	53.9	South Indian children	89.2%/6.2%	6 months
Jellouli (2015)^c^ (18)	Tunisia	R	27	8.7 (mean)	63.0	North African population	NR	Mean, 27.8 months
Ayoob (2016) (28)	United States	R	17	8.0 (mean)	65.0	White, 88%	0%/100%	<1 year
Demircioglu (2018) (29)	Türkiye	R	75	8.2 ± 3.3	56.0	Turkish population	2.7%/97.3%	Until recovery
Gebreyesus (2018) (30)	Ethiopia	R	334	8.6 (mean)	60.5	African children	32.9%/54.2%	During hospitalization
Maalej (2018) (31)	Tunisia	R	178	7.6 ± 3.4	66.7	North African population	11.3%/73.6%	Mean, 9 months
Sepahi (2011) (32)	Iran	R	94	8.2 ± 2.7	55.3	Middle Eastern children	2.1%/97.8%	Mean, 39 months
Bajracharya (2024) (33)	Philippines	R	77	7.8 ± 3.9	57.0	Pediatric tertiary-hospital cohort in the Philippines	NR	During hospitalization
Chong (2023) (34)	Australia	R	96	7.1 (median)	51.0	Aboriginal/Torres Strait Islander, 90.6%	65.6%/27.1%	12 months (poor adherence)
Wan Yusof (2023)^e^ (35)	Malaysia	R	400	7.9 ± 2.9	65.5	Malay, 99%	68.5%/31.5%	Until discharge
Moges (2026) (36)	Ethiopia	R	332	—^d^	63.3	African children	NR/respiratory tract predominant	During hospitalization
Curelaru (2025)^e^ (37)	Israel	R	69	5.0 (median)	∼55.0	Jewish children	NR	Median, 15.5 weeks
Hongsawong (2023) (38)	Thailand	R	96	11.0 (median)	64.6	Southeast Asian children	22.9%/29.2%	Median, 762 days
Dagan (2016) (39)	Israel	R	125	5.8 ± 3.3	74.4	Jewish children	5.6%/44.8%	Mean, 42 months
El-Tigani (2014) (40)	Sudan	R + P	69	9.0 ± 6.6	66.7	African children	NR	1–5 years

R, retrospective cohort; P, prospective cohort; NR, not reported; IQR, interquartile range.

a Age is presented as mean ± standard deviation or median (range/IQR).

b Only the pediatric subgroup (<15 years) was extracted.

c RPGN-specific cohort.

d Only the age range (≤15 years) was reported.

e For these articles, study-year labels indicate first online publication; final issue years are reported in the reference list.

### Study quality assessment

Using the adapted NOS, 12 studies were classified as high quality (7–9 stars), five as moderate quality (5–6 stars), and one as low quality (0–4 stars). Lower scores were mainly associated with biopsy-, RPGN-, or severe/referral-selected cohorts, broader or less specific APSGN ascertainment, and incomplete follow-up or reporting. The detailed item-level NOS assessment is presented in Table 2 and Supplementary Table S8.

**Table 2:** Study quality assessment using the adapted Newcastle-Ottawa scale (NOS).

Study	S1	S2	S3	S4	Comparability (0–2)	O1	O2	O3	Total/quality
Wong (2009) (25)	—	★	★	—	★	★	★	—	5/9 (Moderate)
Becquet (2010) (26)	★	★	★	★	★	★	★	★	8/9 (High)
Luo (2010) (27)	—	★	★	—	★	★	★	★	6/9 (Moderate)
Gunasekaran (2015) (13)	★	★	★	★	★	★	★	★	8/9 (High)
Jellouli (2015) (18)	—	★	★	—	—	★	★	★	5/9 (Moderate)
Ayoob (2016) (28)	—	★	★	—	—	★	★	★	5/9 (Moderate)
Demircioglu (2018) (29)	★	★	★	★	★	★	★	★	8/9 (High)
Gebreyesus (2018) (30)	★	★	—	★	★	★	★	★	7/9 (High)
Maalej (2018) (31)	★	★	★	★	★	★	★	★	8/9 (High)
Sepahi (2011) (32)	—	★	—	★	★	★	★	★	6/9 (Moderate)
Bajracharya (2024) (33)	★	★	★	★	—	★	★	★	7/9 (High)
Chong (2023) (34)	★	★	★	★	★	★	★	★	8/9 (High)
Wan Yusof (2023) (35)	★	★	★	★	★★	★	★	★	9/9 (High)
Moges (2026) (36)	★	★	—	★	★★	★	★	★	8/9 (High)
Curelaru (2025) (37)	★	★	★	★	★	★	★	★	8/9 (High)
Hongsawong (2023) (38)	—	★	—	★	★★	★	★	★	7/9 (High)
Dagan (2016) (39)	—	★	—	★	★★	★	★	★	7/9 (High)
El-Tigani (2014) (40)	—	★	—	—	★	★	★	—	4/9 (Low)

NOS, domains: S1, representativeness; S2, cohort eligibility/source population; S3, APSGN ascertainment; S4, outcome-independent selection; Comparability up to 2 stars; O1, outcome ascertainment; O2, observation window; O3, follow-up/reporting. Quality: high 7–9; moderate 5–6; low 0–4 stars.

### Meta-analysis

Separate single-proportion meta-analyses were conducted for each clinical outcome using the verified event counts and corresponding evaluable sample sizes presented in Table 3. Outcome definitions and evaluable denominators varied across studies. For Jellouli (2015), the exploratory composite used 24 children with crescents among 26 biopsied children, with the denominator restricted to the biopsied subset of the 27-child cohort (18). Among the 10 primary entries in this composite, three studies contributed clinically defined RPGN counts (63 events among 349 children), whereas seven contributed crescentic-lesion counts (45 events among 350 study-level evaluable denominators). Clinical RPGN data were also available for the RPGN-selected Jellouli cohort (27/27), but its primary entry used the histological result (24/26); thus, clinical RPGN counts were available in four studies overall (90 events among 376 children). Separate meta-analyses were not performed because the clinical evidence comprised only four heterogeneous cohorts, including an RPGN-selected cohort, while biopsy-specific denominators were inconsistently reported and biopsied subgroups were selected for severe or atypical disease. The composite therefore cannot estimate the prevalence of either clinical RPGN or crescentic glomerulonephritis separately. Forest plots for hypertension, AKI/acute kidney dysfunction, and RRT are shown in Figures 2–4, with the remaining outcomes presented in Supplementary Figures S1–4.

**Figure 2:**
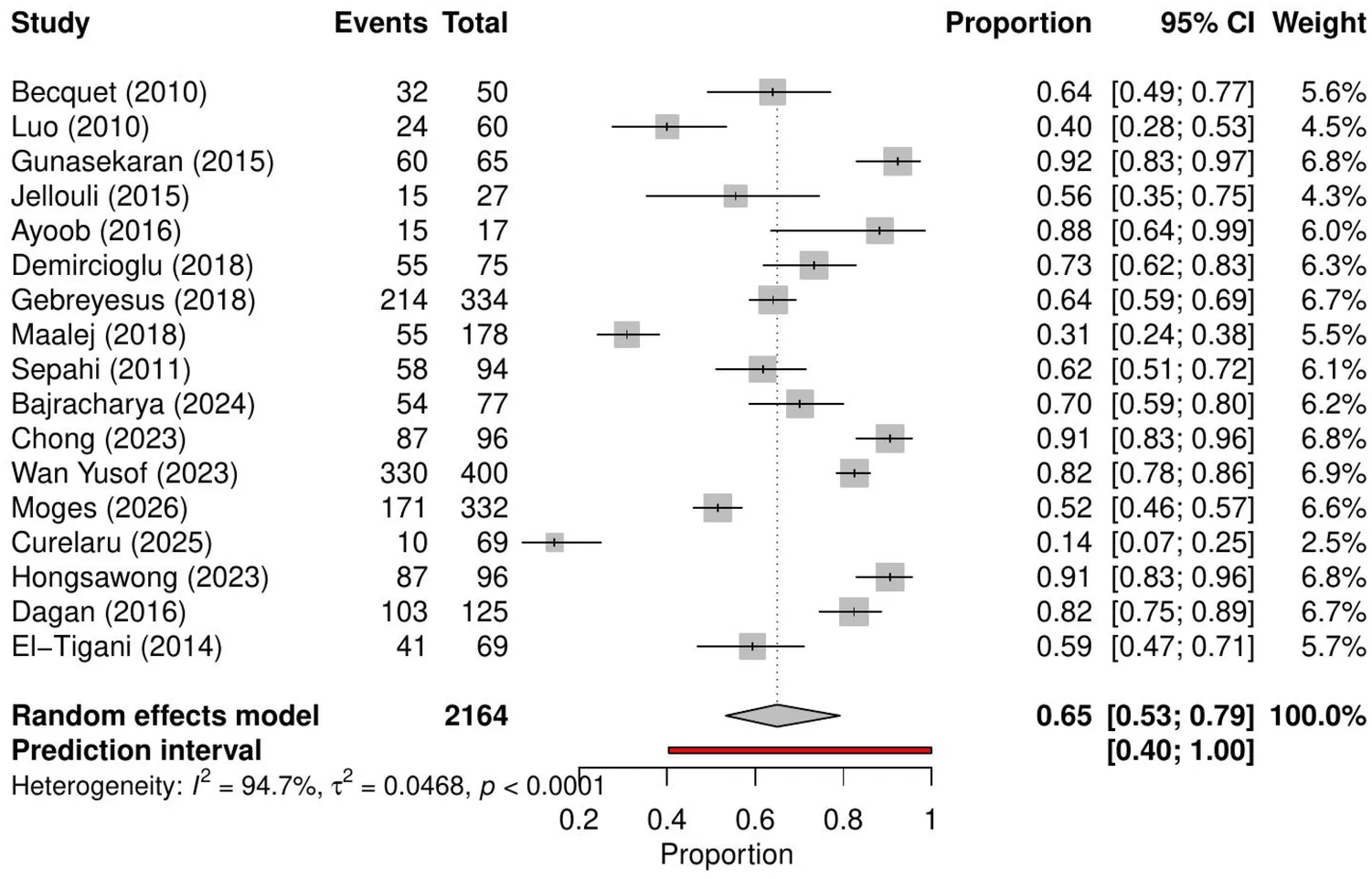
Forest plot of the pooled proportion of hypertension across predominantly hospital-based pediatric APSGN cohorts.

**Figure 3:**
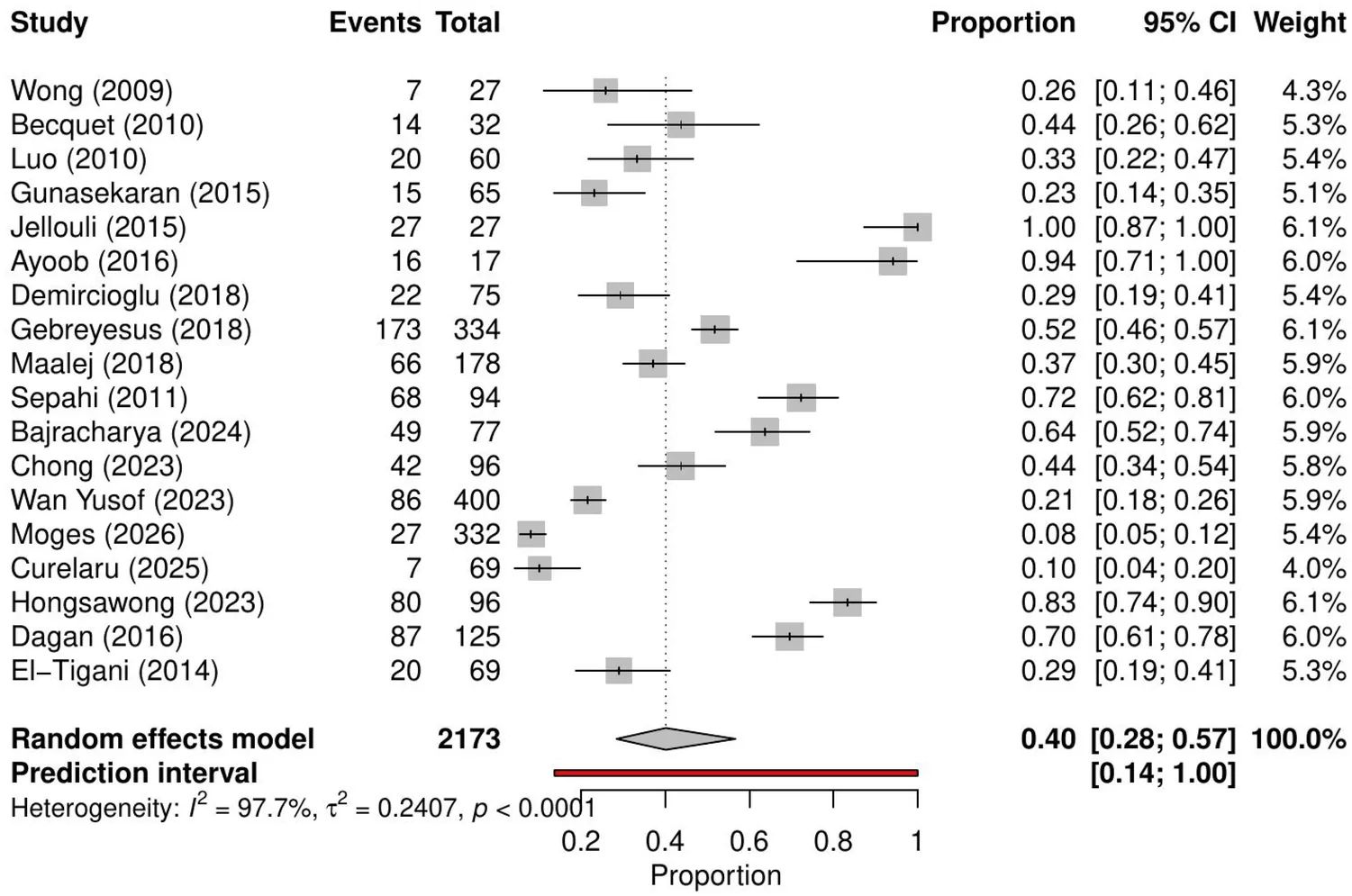
Forest plot of the pooled proportion of study-defined AKI/acute kidney dysfunction across predominantly hospital-based pediatric APSGN cohorts.

**Figure 4:**
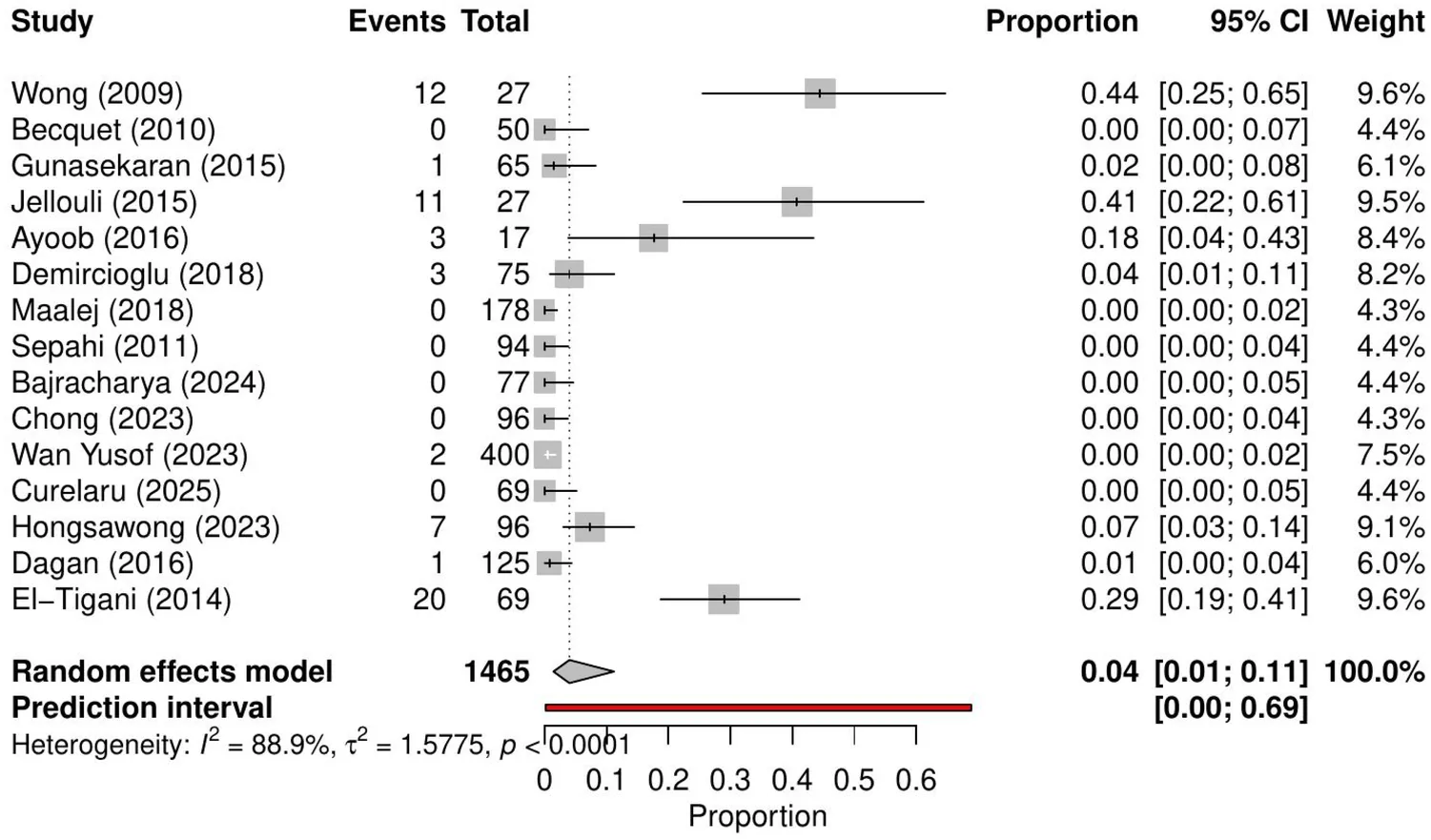
Forest plot of the pooled proportion requiring renal replacement therapy across predominantly hospital-based pediatric APSGN cohorts.

**Table 3:** Summary of seven study-reported core outcome events across the included pediatric cohorts. Operational definitions and evaluable denominators varied across studies; the pooled analyses therefore synthesize study-reported outcome constructs rather than a single standardized definition. For Jellouli (2015), 24/26 denotes crescentic lesions in the biopsied subset of the 27-child cohort; it is not the clinical RPGN event count for the entire cohort. Study-specific outcome definitions and ascertainment details are provided in Supplementary Table S2, and the clinical-RPGN vs. crescentic-lesion contributions to the exploratory composite are detailed in Supplementary Table 2A.

Study (year)	1. Study-defined AKI/acute kidney dysfunction	2. Hypertension	3. Gross hematuria	4. Nephrotic-range proteinuria	5. RRT required	6. RPGN/crescents (exploratory composite)	7. Severe complications
Wong (2009) (25)	7/27	NR	NR	17/27	12/27	11/27	2/27
Becquet (2010) (26)	14/32	32/50	32/50	12/50	0/50	0/50	11/50
Luo (2010) (27)	20/60	24/60	35/60	13/60	NR	NR	0/60
Gunasekaran (2015) (13)	15/65	60/65	40/65	21/65	1/65	1/65	11/65
Jellouli (2015) (18)	27/27	15/27	18/27	11/27	11/27	24/26	2/27
Ayoob (2016) (28)	16/17	15/17	7/17	NR	3/17	3/17	2/17
Demircioglu (2018) (29)	22/75	55/75	62/75	36/75	3/75	11/75	20/75
Gebreyesus (2018) (30)	173/334	214/334	NR	NR	NR	NR	78/334
Maalej (2018) (31)	66/178	55/178	135/178	42/178	0/178	3/178	10/178
Sepahi (2011) (32)	68/94	58/94	65/94	15/94	0/94	NR	1/94
Bajracharya (2024) (33)	49/77	54/77	24/77	NR	0/77	NR	5/77
Chong (2023) (34)	42/96	87/96	NR	52/90	0/96	1/96	33/96
Wan Yusof (2023) (35)	86/400	330/400	121/400	NR	2/400	NR	NR
Moges (2026) (36)	27/332	171/332	47/332	NR	NR	NR	47/332
Curelaru (2025) (37)	7/69	10/69	64/69	NR	0/69	NR	11/69
Hongsawong (2023) (38)	80/96	87/96	37/96	59/96	7/96	49/96	13/96
Dagan (2016) (39)	87/125	103/125	21/125	40/125	1/125	NR	2/125
El-Tigani (2014) (40)	20/69	41/69	NR	NR	20/69	5/69	NR

APSGN, acute post-streptococcal glomerulonephritis; AKI, acute kidney injury; RRT, renal replacement therapy; RPGN, rapidly progressive glomerulonephritis; NR, not reported; GFR, glomerular filtration rate; PCR, protein-to-creatinine ratio; eGFR, estimated GFR; BUN, blood urea nitrogen; Scr, serum creatinine.

Across the predominantly hospital-based and clinically heterogeneous cohorts, hypertension had the highest pooled proportion at 65.0% (17 studies, 2,164 children; 95% CI, 53.3%–79.2%; I^2^ = 94.7%). The pooled proportions of gross hematuria, study-defined AKI/acute kidney dysfunction, and nephrotic-range proteinuria were 47.5% (14 studies, 1,665 children; 95% CI, 34.0%–66.4%; I^2^ = 97.1%), 40.1% (18 studies, 2,173 children; 95% CI, 28.4%–56.7%; I^2^ = 97.7%), and 35.9% (11 studies, 887 children; 95% CI, 26.2%–49.0%; I^2^ = 89.5%), respectively. For severe outcomes, the pooled proportions were 12.9% for severe complications (16 studies, 1,722 children; 95% CI, 8.2%–20.3%; I^2^ = 82.2%), 12.9% for the exploratory RPGN/crescentic-lesion composite (10 studies, 699 evaluable children; 95% CI, 4.1%–40.8%; I^2^ = 95.2%), and 4.0% for RRT (15 studies, 1,465 children; 95% CI, 1.4%–11.2%; I^2^ = 88.9%). Heterogeneity was significant for all outcomes (all *P* < 0.001).

Sensitivity analyses generally supported the pooled estimates for common clinical manifestations, including analyses based on cohort selection, diagnostic specificity, AKI definition, study quality, and leave-one-out exclusion. In contrast, the RRT estimate was more sensitive to cohort selection, decreasing to 2.1% after exclusion of three highly selected cohorts and to 1.3% after exclusion of severe/referral-enriched cohorts. The exploratory RPGN/crescentic-lesion composite decreased from 12.9% overall to 6.1% after exclusion of highly selected cohorts and to 4.6% after exclusion of severe/referral-enriched cohorts; exclusion of broader/less-specific diagnostic cohorts yielded 8.4%. Residual heterogeneity remained substantial. Detailed sensitivity analyses are provided in the Supplementary Material.

### Sensitivity analysis and publication bias

Random-effects leave-one-out estimates for the exploratory RPGN/crescentic-lesion composite ranged from 8.4% to 17.2%, with I^2^ ranging from 91.7% to 95.7%. Omitting Jellouli (2015) yielded 8.6% (95% CI, 2.7%–27.7%), compared with 12.9% overall. These findings indicate sensitivity to study composition and persistent heterogeneity. Detailed leave-one-out results are provided in Supplementary Table S7 and Supplementary Figures S5–11.

In the exploratory AKI definition-restricted analysis, three studies using explicit AKIN or KDIGO criteria comprised 143 AKI events among 561 children and yielded a pooled proportion of 28.1% (95% CI, 10.5%–75.6%; I^2^ = 91.4%), compared with 40.1% in the overall study-defined AKI/acute kidney dysfunction analysis. The very wide confidence interval reflects substantial imprecision as well as between-study heterogeneity; this restricted result should therefore not be regarded as a stable estimate of AKI frequency in pediatric APSGN. Alternative binomial-logit GLMMs produced broadly similar estimates for the common manifestations, whereas the estimate for RRT was lower (1.3% vs. 4.0% in the primary analysis). For the exploratory RPGN/crescentic-lesion composite, the GLMM estimate was 9.7% (95% CI, 1.8%–39.0%), compared with 12.9% in the primary analysis. GLMM-based 95% prediction intervals were wide: 16.5%–95.9% for hypertension, 8.0%–93.9% for gross hematuria, 3.8%–95.1% for study-defined AKI/acute kidney dysfunction, 10.3%–74.4% for nephrotic-range proteinuria, 0.0%–85.3% for RRT, 0.03%–97.1% for the exploratory RPGN/crescentic-lesion composite, and 1.1%–51.4% for study-reported severe complications, indicating substantial between-study variability.

Egger's regression tests indicated statistically significant funnel-plot asymmetry for all seven evaluated outcomes (all *P* < 0.01). Given the limited number of contributing studies (10–18 per outcome), marked clinical and statistical heterogeneity, and the multiple mechanisms that can produce asymmetry, these exploratory results were interpreted only as possible small-study effects and not as evidence that publication bias was present.

## Discussion

This systematic review and meta-analysis included 18 observational studies involving 2,191 children from predominantly hospital-based cohorts. Hypertension was the most frequently reported manifestation, followed by gross hematuria, study-defined AKI/acute kidney dysfunction, and nephrotic-range proteinuria. Severe complications, the exploratory composite of RPGN and/or crescentic lesions, and RRT were reported less frequently. Given the substantial heterogeneity in case ascertainment, clinical setting, disease severity, and outcome definitions, these pooled proportions should be interpreted as summaries of the published clinical cohorts and not directly generalized to broader or unselected pediatric APSGN populations.

Hypertension had the highest pooled proportion in this study. It is primarily attributable to reduced glomerular filtration, sodium and water retention, and expansion of the circulating volume, and is therefore a major focus of monitoring and treatment during the acute phase (7, 9). A nationwide surveillance study in New Zealand reported hypertension in up to 72% of hospitalized children with APSGN; severe cases may also be complicated by acute encephalopathy, heart failure, and pulmonary edema (12). The pooled proportion of severe complications in the present study was 12.9%, indicating that, in addition to hematuria and edema, blood pressure, volume status, neurologic manifestations, and cardiopulmonary findings should be monitored dynamically during the acute phase. The pooled proportion of gross hematuria was close to one-half; however, because some children present only with microscopic hematuria, this estimate may underestimate the overall burden of hematuria (7, 8).

The pooled estimate for kidney involvement reflects study-defined AKI or acute kidney dysfunction rather than a single uniform contemporary AKI definition. Across the included studies, kidney dysfunction was variously operationalized using AKIN or KDIGO criteria, azotemia, age-specific serum creatinine thresholds, reduced eGFR, in-hospital diagnostic criteria, or severe kidney dysfunction requiring dialysis. Consistent with this concern, restricting the analysis to three studies using explicit AKIN or KDIGO definitions (143 events among 561 children) reduced the pooled estimate from 40.1% to 28.1%. However, its very wide 95% CI (10.5%–75.6%) reflects pronounced imprecision in addition to high heterogeneity (I^2^ = 91.4%); accordingly, 28.1% should not be considered a stable estimate of AKI frequency in pediatric APSGN. Thus, differences in AKI definitions likely explain part, but not all, of the observed variability. Clinically, persistent oliguria, progressively rising serum creatinine, or marked proteinuria remains a reason for close monitoring and further evaluation in children with APSGN (9, 10, 16).

RPGN and crescentic lesions were included in the quantitative synthesis as an exploratory study-reported composite, but they should not be regarded as clinically equivalent entities. Clinical RPGN is defined by rapid deterioration in kidney function over a short period, whereas crescentic lesions are histopathological findings that can be identified only in children who undergo kidney biopsy; biopsy itself is generally reserved for severe, atypical, or diagnostically uncertain cases (10, 17, 18). In Jellouli (2015), 24/26 represents crescents among biopsied children and does not estimate clinical RPGN frequency in the full cohort (18). The pooled estimate for this composite is therefore particularly susceptible to outcome misclassification and selection bias and should not be interpreted as the prevalence of either clinical RPGN or crescent formation alone. Separate pooled estimates were not considered valid because clinical RPGN was available in only four heterogeneous cohorts (including the RPGN-selected Jellouli cohort), whereas biopsy-specific denominators were missing or inconsistent in most reports and biopsy was clinically selective. RRT was also strongly affected by case mix: its pooled proportion decreased from 4.0% overall to 2.1% after exclusion of the three highly selected cohorts and to 1.3% after exclusion of severe/referral-enriched cohorts; the alternative GLMM estimate was likewise 1.3%. These findings indicate that rare severe outcomes are more sensitive to referral patterns, disease-severity enrichment, and statistical specification than common clinical manifestations.

Significant between-study heterogeneity was observed for all outcomes, with I^2^ values ranging from 82.2% to 97.7% in the primary analyses. Several factors may have contributed. First, regions differ in the epidemiology of streptococcal infection, socioeconomic conditions, access to health care, and hospitalization criteria (5, 6, 12). Second, the evidence base was predominantly hospital-based, and some studies were conducted in tertiary referral centers or selectively included children with RPGN, kidney biopsy, or severe disease, potentially increasing the observed proportions of kidney dysfunction, RRT, and severe complications (13, 16, 19). Third, diagnostic approaches and operational definitions of AKI, nephrotic-range proteinuria, RPGN/crescentic lesions, and severe complications were not uniform. The wide confidence and prediction intervals indicate substantial uncertainty around several pooled estimates, not merely the presence of statistical heterogeneity, and show that the observed averages are not stable estimates of outcome frequency across pediatric APSGN settings. The wide GLMM-based prediction intervals further emphasized that the expected proportion in a future comparable cohort may differ substantially from the average pooled estimate. Consequently, the pooled proportions should not be generalized directly to community-based or unselected pediatric APSGN populations.

The findings of this study have several implications for clinical practice. During the acute phase of APSGN, blood pressure, urine output, and fluid intake and output should be closely monitored, while serum creatinine, electrolytes, and urinary protein should be assessed serially to facilitate the early identification of volume overload, AKI, and electrolyte disturbances. Rapid deterioration of kidney function, persistent oliguria, difficult-to-control hypertension, marked proteinuria, and prolonged failure of complement levels to normalize may be considered warning signs warranting close observation and further evaluation (9, 10). Although the long-term prognosis of pediatric APSGN is generally favorable and kidney function gradually recovers in most children, a minority may have persistent microscopic hematuria, proteinuria, or borderline hypertension (8, 20). In Australian Indigenous communities with high burdens of streptococcal infection and chronic kidney disease, childhood PSGN has been associated with an increased risk of albuminuria, hematuria, and CKD in adulthood (21–23). These differences suggest that the long-term consequences of APSGN may be jointly influenced by ethnic background, recurrent infection, socioeconomic conditions, and other kidney risk factors. Consequently, children with severe disease during the acute phase and those from high-risk populations may require longer-term follow-up (20–23).

Random-effects leave-one-out analyses supported a more cautious interpretation of the exploratory RPGN/crescentic-lesion composite: estimates ranged from 8.4% to 17.2%, and substantial residual heterogeneity persisted. Funnel-plot asymmetry was statistically significant for all seven outcomes evaluated by Egger's regression. However, with only 10–18 contributing studies per outcome, the reliability of funnel plots and Egger's tests for identifying publication bias is limited. These findings were therefore interpreted only as possible small-study effects, because marked between-study heterogeneity, differences in sample size, referral patterns, disease severity, outcome definitions, study quality, and chance may also produce asymmetry (24). This consideration is particularly important for RRT and the exploratory RPGN/crescentic-lesion composite, which were more sensitive to cohort selection and statistical model specification.

## Strengths

This study has several strengths. First, it systematically integrated clinical data on pediatric APSGN from different countries and regions and explicitly considered the predominantly hospital-based nature of the available evidence. Second, it evaluated both commonly reported manifestations and severe outcomes and rechecked study-level diagnostic approaches and operational outcome definitions to improve transparency. Third, robustness was examined using structured cohort-selection analyses, an AKI definition-restricted analysis, random-effects leave-one-out analyses, alternative binomial-logit GLMMs, and outcome-specific assessment of funnel-plot asymmetry. Fourth, study quality was systematically assessed using an adapted Newcastle-Ottawa Scale (NOS), and the robustness of the main findings was further examined in sensitivity analyses restricted to high-quality studies.

## Limitations

This study also has several limitations. First, most included studies were retrospective, single-center investigations, and incomplete clinical records, selective outcome reporting, and differences in study design may have affected the pooled estimates. Second, substantial heterogeneity was present for all outcomes, while the included cohorts differed in geographic setting, referral pattern, disease severity, diagnostic certainty, and follow-up duration. Third, the evidence base was predominantly hospital-based, and some studies focused on tertiary referral populations, children undergoing kidney biopsy, or children with severe disease; the pooled proportions therefore cannot be generalized directly to all children with APSGN in the community. Fourth, APSGN case ascertainment was not uniform across studies, reflecting differences in the availability and use of streptococcal, complement, clinical, and pathological evidence. Some cohorts permitted alternative evidence pathways or used broader AGN/PIGN definitions. Although study-level criteria were re-evaluated and diagnostic-specificity sensitivity analyses were performed, residual diagnostic misclassification cannot be excluded. Definitions of AKI/acute kidney dysfunction, nephrotic-range proteinuria, RPGN, crescentic lesions, and severe complications also varied across studies. In particular, the combined RPGN/crescentic-lesion analysis is an exploratory composite of distinct clinical and pathological constructs and is vulnerable to biopsy-selection bias. Fifth, denominators differed across outcomes because not every study reported every endpoint and, in some studies, only a subset of participants had evaluable data. Sixth, some studies followed participants only until hospital discharge; thus, this review primarily reflects acute and short-term findings. Seventh, the number of studies was limited for some outcomes, producing wide confidence intervals and substantial imprecision; consequently, those estimates should not be regarded as stable frequencies. Funnel-plot asymmetry may reflect heterogeneity or small-study effects and cannot establish publication bias, particularly with few studies. Finally, although the adapted NOS was used to assess methodological quality, its application to predominantly single-cohort descriptive evidence may not fully capture concerns related to selection, case mix, and heterogeneity in outcome definitions.

Future studies should use standardized APSGN case definitions, AKI staging criteria, and core outcome definitions; adopt multicenter prospective designs encompassing diverse regions and populations; and extend follow-up duration. Severe outcomes, including clinical RPGN, histopathological crescentic lesions, and RRT, should be reported separately. Further studies should also clarify the relationships of persistent proteinuria, severe hypertension, delayed complement recovery, and incomplete recovery of kidney function with long-term kidney outcomes.

## Conclusions

Across the predominantly hospital-based and clinically heterogeneous cohorts, hypertension, gross hematuria, study-defined AKI/acute kidney dysfunction, and nephrotic-range proteinuria were frequently reported, whereas RRT and other severe outcomes were reported less often. The exploratory RPGN/crescentic-lesion composite combined distinct clinical and histopathological constructs and should not be interpreted as a prevalence estimate for either clinical RPGN or crescentic glomerulonephritis separately. Substantial heterogeneity and imprecision limit the stability and generalizability of the pooled estimates; they should not be directly generalized as population-level frequencies for unselected or community-based children with APSGN. Future prospective multicenter studies using standardized case definitions and outcome criteria are needed.

## Data Availability

The original contributions presented in the study are included in the article/Supplementary Material, further inquiries can be directed to the corresponding author/s.

## References

[1] Rodriguez-Iturbe B Musser JM. The current state of post streptococcal glomerulonephritis. J Am Soc Nephrol. (2008) 19(10):1855–64. 10.1681/ASN.2008010092 18667731

[2] Ahn SY Ingulli E. Acute post streptococcal glomerulonephritis: an update. Curr Opin Pediatr. (2008) 20(2):157–62. 10.1097/MOP.0b013e3282f45bcf 18332711

[3] Brant Pinheiro SV De Freitas VB De Castro GV Rufino Madeiro BC De Araújo SA Silva Ribeiro TF. Acute post-streptococcal glomerulonephritis in children: a comprehensive review. Curr Med Chem. (2022) 29(34):5543–59. 10.2174/0929867329666220613103316 35702785

[4] Carapetis JR Steer AC Mulholland EK Weber M. The global burden of group A streptococcal diseases. Lancet Infect Dis. (2005) 5(11):685–94. 10.1016/S1473-3099(05)70267-X 16253886

[5] Marshall CS Cheng AC Markey PG Towers RJ Richardson LJ Fagan PK. Acute post-streptococcal glomerulonephritis in the northern territory of Australia: a review of 16 years data and comparison with the literature. Am J Trop Med Hyg. (2011) 85(4):703–10. 10.4269/ajtmh.2011.11-0185 21976576 PMC3183781

[6] Worthing KA Lacey JA Price DJ McIntyre L Steer AC Tong SYC. Systematic review of group A streptococcal emm types associated with acute post-streptococcal glomerulonephritis. Am J Trop Med Hyg. (2019) 100(5):1066–70. 10.4269/ajtmh.18-0827 30915958 PMC6493963

[7] Eison TM Ault BH Jones DP Chesney RW Wyatt RJ. Post-streptococcal acute glomerulonephritis in children: clinical features and pathogenesis. Pediatr Nephrol. (2011) 26(2):165–80. 10.1007/s00467-010-1554-6 20652330

[8] VanDeVoorde RG 3rd. Acute poststreptococcal glomerulonephritis: the most common acute glomerulonephritis. Pediatr Rev. (2015) 36(1):3–12;quiz 3. 10.1542/pir.36.1.3 25554106

[9] Ong LT. Management and outcomes of acute post-streptococcal glomerulonephritis in children. World J Nephrol. (2022) 11(5):139–45. 10.5527/wjn.v11.i5.139 36187464 PMC9521512

[10] Dhakal AK Shrestha D Preston R Lennon R. Acute post-streptococcal glomerulonephritis in children-treatment standard. Nephrol Dial Transplant. (2025) 40(10):1843–53. 10.1093/ndt/gfaf130 40650562

[11] Meena J Sinha A Krishnasamy S Alba AA Aziz MA Begum A. AsPNA clinical practice guidelines for the management of infection-related glomerulonephritis. Pediatr Nephrol. (2026) 41(6):1867–81. 10.1007/s00467-026-07146-4 41627401

[12] Wong W Lennon DR Crone S Neutze JM Reed PW. Prospective population-based study on the burden of disease from post-streptococcal glomerulonephritis of hospitalised children in New Zealand: epidemiology, clinical features and complications. J Paediatr Child Health. (2013) 49(10):850–5. 10.1111/jpc.12295 23782011

[13] Gunasekaran K Krishnamurthy S Mahadevan S Harish BN Kumar AP. Clinical characteristics and outcome of post-infectious glomerulonephritis in children in southern India: a prospective study. Indian J Pediatr. (2015) 82(10):896–903. 10.1007/s12098-015-1752-0 25893528

[14] Page MJ McKenzie JE Bossuyt PM Boutron I Hoffmann TC Mulrow CD. The PRISMA 2020 statement: an updated guideline for reporting systematic reviews. Syst Rev. (2021) 10(1):89. 10.1186/s13643-021-01626-4 33781348 PMC8008539

[15] Stang A. Critical evaluation of the Newcastle-Ottawa scale for the assessment of the quality of nonrandomized studies in meta-analyses. Eur J Epidemiol. (2010) 25(9):603–5. 10.1007/s10654-010-9491-z 20652370

[16] Ranawaka R Dayasiri K Sandakelum U Nelson D Gamage M. Post-streptococcal acute glomerulonephritis in children: association between proteinuria levels and renal outcomes. World J Clin Pediatr. (2025) 14(1):100885. 10.5409/wjcp.v14.i1.100885 40059899 PMC11686579

[17] Karakaya D Güngör T Çakıcı EK Yazılıtaş F Çelikkaya E Yücebaş SC. Predictors of rapidly progressive glomerulonephritis in acute poststreptococcal glomerulonephritis. Pediatr Nephrol. (2023) 38(9):3027–33. 10.1007/s00467-023-05935-9 36929388

[18] Jellouli M Maghraoui S Abidi K Hammi Y Goucha R Naija O. Outcome of rapidly progressive glomerulonephritis post-streptococcal disease in children. Nephrol Ther. (2015) 11(6):487–91. 10.1016/j.nephro.2015.04.005 26206771

[19] Matsell DG Cojocaru D Catapang M. Risk factors associated with short- and long-term kidney injury in children with post infectious glomerulonephritis. Pediatr Nephrol. (2023) 38(10):3309–15. 10.1007/s00467-023-06000-1 37145185

[20] Clark G White RHR Glasgow EF Chantler C Cameron JS Gill D. Poststreptococcal glomerulonephritis in children: clinicopathological correlations and long-term prognosis. Pediatr Nephrol. (1988) 2(4):381–8. 10.1007/BF00853424 3153045

[21] White AV Hoy WE McCredie DA. Childhood post-streptococcal glomerulonephritis as a risk factor for chronic renal disease in later life. Med J Aust. (2001) 174(10):492–6. 10.5694/j.1326-5377.2001.tb143394.x 11419767

[22] Hoy WE White AV Dowling A Sharma SK Bloomfield H Tipiloura BT. Post-streptococcal glomerulonephritis is a strong risk factor for chronic kidney disease in later life. Kidney Int. (2012) 81(10):1026–32. 10.1038/ki.2011.478 22297679

[23] Hoy WE White AV Tipiloura B Singh GR Sharma S Bloomfield H. The influence of birthweight, past poststreptococcal glomerulonephritis and current body mass index on levels of albuminuria in young adults: the multideterminant model of renal disease in a remote Australian Aboriginal population with high rates of renal disease and renal failure. Nephrol Dial Transplant. (2016) 31(6):971–7. 10.1093/ndt/gfu241 25061125

[24] Sterne JAC Sutton AJ Ioannidis JPA Terrin N Jones DR Lau J. Recommendations for examining and interpreting funnel plot asymmetry in meta-analyses of randomised controlled trials. Br Med J. (2011) 343:d4002. 10.1136/bmj.d4002 21784880

[25] Wong W Morris MC Zwi J. Outcome of severe acute post-streptococcal glomerulonephritis in New Zealand children. Pediatr Nephrol. (2009) 24(5):1021–6. 10.1007/s00467-008-1086-5 19096879

[26] Becquet O Pasche J Gatti H Chenel C Abély M Morville P. Acute post-streptococcal glomerulonephritis in children of French Polynesia: a 3-year retrospective study. Pediatr Nephrol. (2010) 25(2):275–80. 10.1007/s00467-009-1325-4 19876655

[27] Luo C Chen D Tang Z Zhou Y Wang J Liu Z. Clinicopathological features and prognosis of Chinese patients with acute post-streptococcal glomerulonephritis. Nephrology (Carlton). (2010) 15(6):625–31. 10.1111/j.1440-1797.2010.01352.x 20883283

[28] Ayoob RM Schwaderer AL. Acute kidney injury and atypical features during pediatric poststreptococcal glomerulonephritis. Int J Nephrol. (2016) 2016:5163065. 10.1155/2016/5163065 27642522 PMC5011525

[29] Demircioglu Kılıc B Akbalık Kara M Buyukcelik M Balat A. Pediatric post-streptococcal glomerulonephritis: clinical and laboratory data. Pediatr Int. (2018) 60(7):645–50. 10.1111/ped.13587 29729114

[30] Gebreyesus LG Aregay AF Gebrekidan KG Alemayehu YH. Factors associated with treatment outcome of acute post streptococcal glomerulonephritis among patients less than 18 years in mekelle city, public hospitals, north Ethiopia. BMC Res Notes. (2018) 11(1):693. 10.1186/s13104-018-3794-7 30285899 PMC6167782

[31] Maalej B Ben Amor M Jallouli M Weli M Charfi M Abdelkefi Y. Post-streptococcal glomerulonephritis in the south of Tunisia: a 12-year retrospective review. Nephrol Ther. (2018) 14(7):518–22. 10.1016/j.nephro.2018.04.001 29735420

[32] Sepahi MA Shajari A Shakiba M Shooshtary FK Salimi MH. Acute glomerulonephritis: a 7 years follow up of children in center of Iran. Acta Med Iran. (2011) 49(6):375–8. 21874641

[33] Bajracharya P Khadgi A Shrestha S Silwal R Tandukar A. Acute post-streptococcal glomerulonephritis in a pediatric population: a five-year retrospective study. Cureus. (2024) 16(3):e56082. 10.7759/cureus.56082 38618409 PMC11009898

[34] Chong HC Hung TY Hohls A Francis JR Chaturvedi S. Clinical characteristics of hospitalised children with acute post-streptococcal glomerulonephritis in the top end of Australia. J Paediatr Child Health. (2023) 59(5):735–42. 10.1111/jpc.16386 36975716

[35] Wan Yusof WA Yaacob NM Nasir A Yusoff S Ilias MI. Clinical predictors of acute kidney injury in children with acute post-streptococcal glomerulonephritis: a tertiary centre experience. Singapore Med J. (2025) 66(1):54–7. 10.4103/singaporemedj.SMJ-2021-092 36695277 PMC11809744

[36] Moges TA Dagnew SB Yayeh YT Wondm SA Dagnew FN Tarekegn GY. Outcomes and predictors of acute post-streptococcal glomerulonephritis in hospitalized children in northwest Ethiopia: a multicenter retrospective cohort study. Health Sci Rep. (2026) 9(7):e72751. 10.1002/hsr2.72751 42382504 PMC13316804

[37] Curelaru S Gelman E Lebel A. Fever in acute post-streptococcal glomerulonephritis: prevalence and clinical significance. J Pediatr Health Care. (2026) 40(4):603–7. 10.1016/j.pedhc.2025.09.002 41065602

[38] Hongsawong N Suwansirikul S Chartapisak W. Clinical manifestations, pathological correlations, prognostic factors, and outcomes of severe acute postinfectious glomerulonephritis with rapidly progressive glomerulonephritis in children. J Med Assoc Thail. (2023) 106(3):287. 10.35755/jmedassocthai.2023.03.13805

[39] Dagan R Cleper R Davidovits M Sinai-Trieman L Krause I. Post-infectious glomerulonephritis in pediatric patients over two decades: severity-associated features. Isr Med Assoc J. (2016) 18(6):336–40. 27468526

[40] Ali el TM Babikir AM El-Assad S Abdelrahim MB. Prognosis of acute post-streptococcal glomerulonephritis in Sudanese children. Arab J Nephrol Transplant. (2014) 7(2):103–7. 25366505

