# Supplementary material for: Clinical manifestations and proportions of severe outcomes in pediatric acute post-streptococcal glomerulonephritis: a systematic review and meta-analysis of predominantly hospital-based cohorts
Source: Front Med (Lausanne). 2026 Sep 25;13:1953518. doi: 10.3389/fmed.2026.1953518 (PMC13649450; doi:10.3389/fmed.2026.1953518)
Supplement: Supplementary file 1 [file Supplementaryfile1.docx]

**SUPPLEMENTARY MATERIALS**

**Clinical Manifestations and Proportions of Severe Outcomes in Pediatric Acute Post-Streptococcal Glomerulonephritis: A Systematic Review and Meta-Analysis of Predominantly Hospital-Based Cohorts**

PROSPERO registration: CRD420261464457

**Supplementary Methods: Complete Search Strategy**

PubMed, Cochrane Library, Web of Science, Embase, Scopus, and ClinicalTrials.gov were searched from database inception through July 15, 2026. The original search used a sensitive, predominantly free-text strategy adapted to the title, abstract, and keyword fields available in each database. It combined direct disease-specific terminology with a decomposed streptococcal-plus-glomerulonephritis concept strategy. Database-specific controlled-vocabulary headings were not systematically required components of the original searches. No language restrictions were applied. Reference lists of included studies were manually screened. The line-by-line strategies below reproduce the searches used for the review; they are provided here rather than repeated in the main manuscript.

**PubMed**

| **No.** | **Query** | **Results** |
| --- | --- | --- |
| 1 | Streptococcal | 23795 |
| 2 | (((Glomerulonephritides[Title/Abstract]) OR (Kidney Scarring[Title/Abstract])) OR (Scarring, Kidney[Title/Abstract])) OR (Bright Disease[Title/Abstract]) | 588 |
| 3 | (post-streptococcal glomerulonephritis[Title/Abstract]) OR (PSGN[Title/Abstract]) | 536 |
| 4 | 1 AND 2 | 16 |
| 5 | 4 OR 3 | 544 |

**Cochrane Library**

| **No.** | **Query** | **Results** |
| --- | --- | --- |
| 1 | Streptococcal:ab,ti,kw | 1349 |
| 2 | (Glomerulonephritides OR 'Kidney Scarring' OR 'Scarring, Kidney' OR 'Bright Disease'):ab,ti,kw | 518 |
| 3 | ('post-streptococcal glomerulonephritis' OR PSGN):ab,ti,kw | 13 |
| 4 | 1 AND 2 | 0 |
| 5 | 4 OR 3 | 13 |

**Web of Science**

| **No.** | **Query** | **Results** |
| --- | --- | --- |
| 1 | TS=Streptococcal | 8996 |
| 2 | TS=(Glomerulonephritides OR 'Kidney Scarring' OR 'Scarring, Kidney' OR 'Bright Disease') | 24406 |
| 3 | TS=('post-streptococcal glomerulonephritis' OR PSGN) | 257 |
| 4 | 1 AND 2 | 355 |
| 5 | 4 OR 3 | 378 |

**Embase**

| **No.** | **Query** | **Results** |
| --- | --- | --- |
| 1 | Streptococcal:ab,ti,kw | 27462 |
| 2 | Glomerulonephritides:ab,ti,kw OR 'Kidney Scarring':ab,ti,kw OR 'Scarring, Kidney':ab,ti,kw OR 'Bright Disease':ab,ti,kw | 821 |
| 3 | 'post-streptococcal glomerulonephritis':ab,ti,kw OR PSGN:ab,ti,kw | 744 |
| 4 | 1 AND 2 | 21 |
| 5 | 4 OR 3 | 756 |

**Scopus**

| **No.** | **Query** | **Results** |
| --- | --- | --- |
| 1 | TITLE-ABS-KEY (Streptococcal) | 53105 |
| 2 | TITLE-ABS-KEY (Glomerulonephritides OR 'Kidney Scarring' OR 'Scarring, Kidney' OR 'Bright Disease') | 2258 |
| 3 | TITLE-ABS-KEY ('post-streptococcal glomerulonephritis' OR PSGN) | 710 |
| 4 | 1 AND 2 | 5 |
| 5 | 4 OR 3 | 714 |

**ClinicalTrials.gov**

| **No.** | **Query** | **Results** |
| --- | --- | --- |
| 1 | "post-streptococcal glomerulonephritis" OR PSGN | 2 |

**Supplementary Tables**

**Supplementary Table S1. Diagnostic ascertainment and cohort-selection characteristics of included studies**

| **Study** | **Reported diagnostic ascertainment and role of preceding streptococcal evidence** | **Setting / selection features** | **Diagnostic interpretation** |
| --- | --- | --- | --- |
| Wong (2009) | Diagnostic pathway: Streptococcal evidence included in the stated criteria; uniform documentation not demonstrated. The criteria included elevated streptococcal titres, transiently depressed C3, absence of prior chronic kidney disease, biopsy findings, and a compatible clinical course. Elevated streptococcal titres were reported in 18/27 children, and C3 was reduced in 16/26 tested children. | Tertiary referral; all patients identified through renal biopsy; biopsy indications included anuric/severe renal failure, mixed nephritic-nephrotic syndrome, or delayed recovery. | High diagnostic support, but strongly biopsy/severity selected. |
| Becquet (2010) | Diagnostic pathway: Preceding streptococcal evidence explicitly required. Hematuria, decreased C3, and evidence of recent streptococcal infection; ASO and/or anti-DNase B thresholds were specified. | Hospitalized children in French Polynesia. | Relatively explicit APSGN definition. |
| Luo (2010) | Diagnostic pathway: Preceding streptococcal evidence explicitly required. Hematuria/proteinuria, recent group A streptococcal evidence by ASO, anti-DNase B, or throat culture, hypocomplementemia, and endocapillary proliferative glomerulonephritis on pathology. | Patients identified from kidney biopsy specimens; pediatric subgroup extracted. | High diagnostic certainty but biopsy selected. |
| Gunasekaran (2015) | Diagnostic pathway: Preceding streptococcal evidence explicitly required. PSGN required hematuria/proteinuria, clinical-serological evidence of recent streptococcal infection, and low C3 with normalization at 8 weeks. | Prospective tertiary-care cohort of acute nephritic syndrome; PSGN subgroup (n=65) separately reported. | Explicit PSGN subgroup available. |
| Jellouli (2015) | Diagnostic pathway: Preceding streptococcal evidence explicitly required. The diagnosis was retained in children with a recent streptococcal infection, elevated antibodies against streptococcal antigens, and a transient decrease in C3; the study then selected children with worsening kidney function/RPGN. | RPGN-specific severe-disease cohort. | Highly selected severe/RPGN population. |
| Ayoob (2016) | Diagnostic pathway: Preceding streptococcal evidence explicitly required. Hematuria, low C3 that normalized, and laboratory documentation of streptococcal infection or household contact; other renal diseases were excluded. | Pediatric tertiary-care center; inpatient and outpatient cases; case series enriched for severe/atypical presentations. | Explicit criteria but referral/severity enrichment. |
| Demircioglu (2018) | Diagnostic pathway: Streptococcal evidence included in the stated criteria, with pathological confirmation used in one case. The criteria included hematuria, decreased C3, and recent streptococcal infection established by elevated ASO; one ASO-negative child was confirmed by kidney biopsy. | Pediatric nephrology clinic. | Explicit APSGN definition. |
| Gebreyesus (2018) | Diagnostic pathway: Uniform algorithm not fully described. Medical records coded/diagnosed as APSGN were included; ASO was measured in 68/334 children. | Hospital-based retrospective study in Ethiopia. | Diagnostic certainty less completely reported. |
| Maalej (2018) | Diagnostic pathway: Preceding streptococcal evidence explicitly required. Hematuria with or without proteinuria, ASO >200 IU/L as evidence of recent streptococcal infection, transient C3 reduction, and no alternative glomerular disease. | Hospitalized pediatric cohort. | Explicit APSGN definition. |
| Sepahi (2011) | Diagnostic pathway: Broader cohort-level ascertainment. AGN was diagnosed clinically using preceding streptococcal infection and supportive laboratory findings, while outcomes were reported for the overall 94-child AGN cohort, which included 87 APSGN cases and seven other diagnoses. | Hospitalized AGN cohort; outcomes reported for the overall AGN cohort in the article. | Broader AGN cohort; interpret diagnostic specificity cautiously. |
| Bajracharya (2024) | Diagnostic pathway: Multiple evidence combinations permitted. The report used confirmed and probable case categories; probable cases could be classified from clinical evidence alone, whereas confirmed cases required biopsy evidence or combined clinical and laboratory evidence. | Hospitalized tertiary-care pediatric cohort. | Explicit case verification described. |
| Chong (2023) | Diagnostic pathway: Multiple evidence combinations permitted. The Northern Territory case definition allowed confirmed, probable, and possible cases; a probable case could meet clinical evidence alone, whereas suggestive laboratory evidence included hematuria, streptococcal evidence, and reduced C3. | Hospitalized children at a tertiary hospital; surveillance-linked case ascertainment. | Standardized case definition; hospital-based. |
| Wan Yusof (2023) | Diagnostic pathway: Streptococcal evidence not uniformly documented. Diagnosis by the treating pediatrician used clinical findings together with low C3 and/or recent streptococcal evidence; ASO was positive in 163/400 children. | All admitted APSGN children at a tertiary teaching hospital. | Hospital-based explicit clinical/laboratory approach. |
| Moges (2026) | Diagnostic pathway: Streptococcal evidence included in the stated criteria; uniform documentation not demonstrated. The operational definition required nephritic clinical evidence plus microscopic hematuria, recent streptococcal evidence (culture, ASO, or anti-DNase B), and reduced C3; ASO was measured in 73/332 children, while uniform use of the alternative tests was not reported. | Multicenter hospitalized cohort in Northwest Ethiopia. | Detailed diagnostic criteria reported. |
| Curelaru (2025) | Diagnostic pathway: Preceding streptococcal evidence explicitly required. Inclusion required clinical glomerulonephritis findings, low C3, and ASLO >200 IU/mL; other glomerulonephritides and cases without follow-up were excluded. | Single-center hospitalized cohort. | Explicit clinical/laboratory definition. |
| Hongsawong (2023) | Diagnostic pathway: Multiple evidence combinations permitted. PIGN was defined by acute glomerulonephritis with hematuria/proteinuria plus transient low C3, evidence of previous infection, or compatible pathology; preceding streptococcal evidence was one of several accepted elements. | Tertiary hospital PIGN cohort; patients stratified by clinical RPGN. | Broader PIGN definition; not uniformly restricted to streptococcal evidence. |
| Dagan (2016) | Diagnostic pathway: Multiple evidence combinations permitted. PIGN criteria comprised nephritic findings with hypocomplementemia and/or microbiological or serological streptococcal evidence; the authors reported probable non-streptococcal triggers in a few children. | Hospitalized pediatric PIGN cohort. | Broader PIGN cohort; diagnostic specificity varies. |
| El-Tigani (2014) | Diagnostic pathway: Composite clinical criteria. APSGN was based on any two or more of hematuria, edema, hypertension, and elevated/rising ASO as evidence of antecedent streptococcal infection; ASO was therefore one component of the diagnostic combination rather than a mandatory item in every case. | National tertiary referral pediatric renal unit; high proportion of severe cases requiring dialysis. | Hospital/referral cohort with variable laboratory confirmation. |

Note: These classifications describe how evidence entered the diagnostic pathway reported by each primary study; they do not imply that individual cases were retrospectively reclassified as invalid. Seven studies explicitly required preceding streptococcal evidence without a reported alternative diagnostic route. In three, such evidence appeared in the stated criteria but was not documented uniformly across all records. Seven used broader or alternative combinations of clinical, laboratory, or pathological evidence, and one did not describe a uniform algorithm in sufficient detail. These differences reflect the original case definitions and availability of evidence in the primary reports; no additional uniform requirement was imposed retrospectively by this review. Evidence included ASO/ASLO, anti-DNase B, throat or skin culture/swab, and compatible clinical or epidemiologic history. Nephritic features and hypocomplementemia alone were not treated by this review as proof of preceding streptococcal infection. ASO/ASLO, antistreptolysin O; C3, complement component 3; AGN, acute glomerulonephritis; PIGN, postinfectious glomerulonephritis.

**Supplementary Table S2. Study-specific operational definitions relevant to the pooled outcomes**

| **Study** | **AKI / acute kidney dysfunction** | **Hypertension** | **Nephrotic-range proteinuria** | **RPGN / crescents** | **Severe complications / notes** |
| --- | --- | --- | --- | --- | --- |
| Wong (2009) | Severe renal dysfunction in a biopsy-selected cohort; the reported event count was used. | NR for pooled HTN outcome. | 4+ dipstick protein or urine protein/creatinine ratio >200 mg/mmol. | Crescents quantified on biopsy; cohort divided by >=50% vs <50% crescents. | Biopsy indications themselves reflected severe/atypical disease. |
| Becquet (2010) | Renal failure: GFR <80 mL/min/1.73 m2 (subset with evaluable GFR). | Hypertension reported; severe hypertension >97.5th percentile +30 mmHg. | Protein/creatinine ratio >3 g/g. | No crescents in the biopsied severe-proteinuria case; a zero-event count was used. | Severe presentation: heart failure, severe hypertension and/or encephalopathy. |
| Luo (2010) | Study-reported kidney dysfunction/renal impairment; the reported event count was used. | Study-reported. | Study-reported; the reported event count was used. | Pathology was available because all patients were biopsy-confirmed; the crescent count was not reported for pooling. | The study-reported severe-complication count was used. |
| Gunasekaran (2015) | AKIN classification; pRIFLE also reported. | Study-reported pediatric hypertension; hypertensive emergency separately reported. | UPCR >2 g/g. | Biopsy in suspected RPGN; crescentic GN defined histopathologically. | Heart failure, encephalopathy, hypertensive emergency and retinopathy reported separately. |
| Jellouli (2015) | All patients had renal failure in an RPGN-specific cohort. | Study-reported. | Nephrotic syndrome/proteinuria reported. | Clinical RPGN was the cohort-defining condition; crescents occurred in 24 of 26 biopsied children. The exploratory composite uses 24/26, not the clinical RPGN event count for the 27-child cohort. | Dialysis and adverse kidney outcomes common; highly selected cohort. |
| Ayoob (2016) | Study-reported AKI with eGFR calculated using Schwartz; cohort had high frequency of reduced GFR. | Study-reported. | NR. | Four severe/atypical patients biopsied; cellular crescents in three. | Severe/atypical manifestations included dialysis, seizures, thrombocytopenia. |
| Demircioglu (2018) | The pooled event count corresponds to GFR <90 mL/min/1.73 m2. | Study-reported hypertension. | UPCR >2 or >40 mg/m2/h. | The extracted count of 11/75 refers to clinical RPGN. Kidney biopsy was performed in 19 patients; seven of the 11 patients with elevated serum creatinine had cellular crescents involving 10–20% of glomeruli. | Pulmonary edema/heart failure and PRES reported. |
| Gebreyesus (2018) | The pooled event count uses azotemia/elevated BUN or renal-function abnormality as reported. | Reported as a complication; threshold not clearly specified in the article. | NR. | NR. | Encephalopathy, heart failure, pulmonary edema reported as complications. |
| Maalej (2018) | Serum creatinine >=70 micromol/L defined acute renal failure. | SBP and/or DBP >97.5th percentile for height and sex. | Nephrotic syndrome definition included proteinuria >=50 mg/kg/day or UPCR >2 g/g with hypoalbuminemia. | RPGN: progressive acute renal failure over days/weeks associated with a high percentage of epithelial crescents. | Cardiovascular, pleuropulmonary and neurologic complications reported. |
| Sepahi (2011) | Azotemia: BUN >20 mg/dL in the AGN cohort. | Study-reported. | Nephrotic protein excretion >40 mg/m2/h. | Biopsy indicated for RPGN or persistent abnormalities; pooled table has NR. | Hypertensive encephalopathy reported. |
| Bajracharya (2024) | Acute renal injury based on age-specific normal creatinine values. | Clinical APSGN definition included hypertension; study reported hospital hypertension. | NR. | NR. | Pleural effusion and hypertensive encephalopathy reported. |
| Chong (2023) | 2012 KDIGO AKI criteria, including serum creatinine and urine-output staging. | 2017 AAP pediatric BP criteria. | PCR >200 mg/mmol. | Biopsy performed only for worsening/atypical cases; one crescentic APSGN case reported. | Hypertensive emergencies and other severe inpatient events reported. |
| Wan Yusof (2023) | KDIGO-based AKI; because baseline creatinine/oliguria were often unavailable, age-specific upper-limit creatinine increase was used operationally. | SBP/DBP >95th percentile for age, height, and sex. | NR for the pooled analysis. | NR. | Severe AKI requiring hemodialysis reported separately. |
| Moges (2026) | In-hospital AKI diagnosis as reported in the study. | Reported as a hospital complication. | NR. | NR. | Encephalopathy, heart failure, pulmonary edema, and other complications reported separately. |
| Curelaru (2025) | eGFR calculated by Schwartz; eGFR <90 mL/min/1.73 m2 was considered abnormal, and the reported event count was used. | SBP or DBP >95th percentile for age, sex, and height. | NR. | NR. | Pulmonary congestion/edema and PRES reported. |
| Hongsawong (2023) | Study-defined kidney dysfunction assessed using eGFR; the extracted count is 80/96. This is distinct from the clinical RPGN outcome. | BP categorized as elevated/stage 1/stage 2/hypertensive crisis. | UPCR >2 or >40 mg/m2/h. | Clinical RPGN defined as eGFR decline >=50% over <=3 months; the extracted count is 49/96. Pathology was available only in a selected biopsied subgroup. | KRT and severe RPGN-related outcomes reported. |
| Dagan (2016) | The pooled event count uses azotemia as reported. | BP >95th percentile for age, sex, and height. | Nephritic-nephrotic presentation was reported; the reported event count was used. | Biopsy only in selected cases; pooled table has NR. | Hemodialysis and hypertensive encephalopathy were rare. The extracted gross-hematuria count (21/125) represents isolated gross hematuria. |
| El-Tigani (2014) | The pooled event count represents severe acute kidney failure requiring dialysis. | >95th percentile for age and gender. | NR for the pooled analysis. | Crescent formation assessed only among biopsied severe/atypical cases. | Tertiary-referral cohort; 29% required dialysis at presentation. |

**Supplementary Table S2A. Clinical RPGN and histopathological crescentic-lesion data contributing to the exploratory composite**

| **Study** | **Primary composite construct and entry** | **Other relevant reported data** | **Denominator and interpretation** |
| --- | --- | --- | --- |
| Wong (2009) | Crescentic lesions: 11/27 | Clinical RPGN count not separately reported | All 27 children were identified through kidney biopsy; biopsy/severity-selected cohort. |
| Becquet (2010) | Crescentic lesions: 0/50 | No crescent in the one biopsied severe-proteinuria case | The composite denominator is the cohort denominator, not a biopsy-prevalence denominator. |
| Gunasekaran (2015) | Crescentic lesion/crescentic GN: 1/65 | Biopsy was undertaken for suspected RPGN; biopsy denominator not consistently extractable | Cohort-level entry; not an estimate among all biopsied children. |
| Jellouli (2015) | Crescentic lesions: 24/26 | Clinical RPGN: 27/27 (cohort-defining condition) | The primary composite entry uses 24/26 biopsied children rather than 27/27 clinical RPGN. |
| Ayoob (2016) | Crescentic lesions: 3/17 | Three of four biopsied severe/atypical children had cellular crescents | The composite denominator is the full cohort; biopsy was clinically selected. |
| Demircioglu (2018) | Clinical RPGN: 11/75 | Cellular crescents were described in 7 of 19 biopsied children | The primary composite entry is clinical RPGN, not the histological count. |
| Maalej (2018) | Clinical RPGN: 3/178 | Separate biopsy-specific crescent denominator not extractable | Clinical RPGN was defined by rapidly progressive renal failure with extensive crescents. |
| Chong (2023) | Crescentic lesion: 1/96 | One crescentic APSGN case was reported | Cohort-level entry; biopsy was reserved for worsening/atypical cases. |
| Hongsawong (2023) | Clinical RPGN: 49/96 | Pathology was available only in a selected biopsied subgroup | The primary composite entry is the prespecified clinical RPGN definition (>=50% eGFR decline within <=3 months). |
| El-Tigani (2014) | Crescentic lesions: 5/69 | Biopsy-specific denominator not consistently extractable | Cohort-level entry from a severe tertiary-referral population; biopsy was selective. |

Note: The 10 primary composite entries partition into three clinically defined RPGN entries (63 events/349 children) and seven crescentic-lesion entries (45 events/350 study-level evaluable denominators). Clinical RPGN data were available in four studies overall (90 events/376 children) because Jellouli (2015) also reported 27/27 clinical RPGN, while its primary composite entry used 24/26 biopsy-detected crescents. Separate pooled estimates were not generated because the clinical evidence was sparse and included an RPGN-selected cohort, while biopsy-specific denominators were missing or inconsistent in most histopathological reports and biopsy was clinically selective. Therefore, the 12.9% exploratory composite must not be interpreted as the prevalence of either clinical RPGN or crescentic glomerulonephritis separately.

**Supplementary Table S3. Structured sensitivity analyses of cohort selection and diagnostic specificity**

| **Outcome** | **Overall analysis** | **Exclude highly selected cohorts** | **Exclude severe/referral-enriched cohorts** | **Exclude broader/less-specific diagnostic cohorts** |
| --- | --- | --- | --- | --- |
| Study-defined AKI/acute kidney dysfunction | 40.1% (28.4%-56.7%); I²=97.7%; k=18, N=2,173 | 38.9% (26.3%-57.5%); I²=97.0%; k=15, N=2,059 | 36.7% (24.7%-54.4%); I²=96.8%; k=14, N=2,033 | 34.4% (23.4%-50.5%); I²=98.1%; k=15, N=1,858 |
| Hypertension | 65.0% (53.3%-79.2%); I²=94.7%; k=17, N=2,164 | 67.2% (54.4%-83.1%); I²=95.0%; k=15, N=2,077 | 64.5% (50.8%-81.8%); I²=95.5%; k=14, N=2,051 | 61.6% (48.3%-78.6%); I²=95.2%; k=14, N=1,849 |
| Gross hematuria | 47.5% (34.0%-66.4%); I²=97.1%; k=14, N=1,665 | 45.3% (30.6%-67.1%); I²=97.5%; k=12, N=1,578 | 46.6% (31.5%-68.9%); I²=97.5%; k=12, N=1,621 | 50.9% (34.9%-74.1%); I²=97.3%; k=11, N=1,350 |
| Nephrotic-range proteinuria | 35.9% (26.2%-49.0%); I²=89.5%; k=11, N=887 | 34.7% (23.4%-51.4%); I²=91.4%; k=8, N=773 | 33.1% (23.1%-47.5%); I²=91.0%; k=9, N=833 | 37.0% (25.9%-52.7%); I²=87.0%; k=8, N=572 |
| RRT | 4.0% (1.4%-11.2%); I²=88.9%; k=15, N=1,465 | 2.1% (0.8%-5.4%); I²=86.4%; k=13, N=1,411 | 1.3% (0.6%-2.7%); I²=56.7%; k=11, N=1,325 | 4.9% (1.4%-16.9%); I²=88.7%; k=12, N=1,150 |
| Exploratory RPGN/crescentic-lesion composite | 12.9% (4.1%-40.8%); I²=95.2%; k=10, N=699 | 6.1% (1.7%-21.3%); I²=92.7%; k=8, N=646 | 4.6% (0.8%-27.6%); I²=93.8%; k=6, N=560 | 8.4% (2.3%-30.2%); I²=95.5%; k=9, N=603 |
| Study-reported severe complications | 12.9% (8.2%-20.3%); I²=82.2%; k=16, N=1,722 | 14.1% (8.6%-23.0%); I²=84.1%; k=13, N=1,608 | 13.5% (7.9%-23.3%); I²=85.0%; k=13, N=1,651 | 15.3% (10.2%-22.9%); I²=80.3%; k=13, N=1,407 |

Note: Sensitivity analyses used the final verified extraction (Jellouli [2015], 24/26 for crescentic lesions among biopsied children). Analyses excluding Jellouli were unaffected by this event-definition choice. 'Highly selected cohorts' = Wong (2009), Luo (2010), and Jellouli (2015). 'Severe/referral-enriched cohorts' = Wong (2009), Jellouli (2015), Ayoob (2016), and El-Tigani (2014). Diagnostic-specificity sensitivity excluded Sepahi (2011), Hongsawong (2023), and Dagan (2016). Because biopsy-selected, RPGN-specific, and severe/referral characteristics overlapped across several cohorts and some categories contained only one or two studies, separate pooled subgroup estimates for each selection label were not considered sufficiently stable; the structured exclusion sets were used to assess their combined influence.

**Supplementary Table S4. AKI definition-restricted sensitivity analysis**

| **Analysis** | **No. studies** | **Pooled proportion (95% CI)** | **I²** | **Events / participants** |
| --- | --- | --- | --- | --- |
| Overall study-defined AKI/acute kidney dysfunction | 18 | 40.1% (28.4%-56.7%) | 97.7% | 826/2,173 |
| Restricted to explicit AKIN/KDIGO definitions | 3 | 28.1% (10.5%-75.6%) | 91.4% | 143/561 |

Note: The definition-restricted analysis included Gunasekaran (2015), Chong (2023), and Wan Yusof (2023). It was exploratory because only three studies used explicit contemporary AKI frameworks.

**Supplementary Table S5. Alternative binomial-logit GLMM sensitivity analyses and prediction intervals**

| **Outcome** | **k** | **GLMM pooled proportion (95% CI)** | **95% prediction interval** | **I²** | **N** |
| --- | --- | --- | --- | --- | --- |
| Study-defined AKI/acute kidney dysfunction | 18 | 46.8% (29.7%-64.6%) | 3.81%-95.1% | 95.4% | 2,173 |
| Hypertension | 17 | 68.3% (54.7%-79.3%) | 16.54%-95.9% | 94.7% | 2,164 |
| Gross hematuria | 14 | 53.7% (37.0%-69.6%) | 8.02%-93.9% | 96.3% | 1,665 |
| Nephrotic-range proteinuria | 11 | 36.6% (26.1%-48.6%) | 10.29%-74.4% | 88.9% | 887 |
| RRT | 15 | 1.3% (0.2%-7.5%) | 0.00%-85.3% | 83.7% | 1,465 |
| Exploratory RPGN/crescentic-lesion composite | 10 | 9.7% (1.8%-39.0%) | 0.03%-97.1% | 92.4% | 699 |
| Study-reported severe complications | 16 | 9.8% (5.6%-16.7%) | 1.10%-51.4% | 81.4% | 1,722 |

Note: Random-intercept binomial-logit GLMMs were used as alternative-model sensitivity analyses. These models do not require continuity corrections for zero-event studies and provide prediction intervals constrained to the 0–100% proportion scale. Prediction intervals displayed in the primary forest plots are based on the primary inverse-variance random-effects model, whereas the bounded intervals presented in this table are derived from the alternative GLMM sensitivity analysis and therefore are not expected to be numerically identical. For the exploratory RPGN/crescentic-lesion composite, the unbounded primary-model 95% prediction interval was 1.41%–117.78%; values above 100% reflect the limitations of the PLN model. The upper prediction limit is capped at 100% for display in Figures S3 and S10, whereas the bounded GLMM interval is 0.03%–97.1%.

**Supplementary Table S6. Outcome-specific Egger tests for funnel-plot asymmetry**

| **Outcome** | **k** | **Egger statistic** | **P value** | **Interpretation** |
| --- | --- | --- | --- | --- |
| Study-defined AKI/acute kidney dysfunction | 18 | -5.547 | <0.001 | Possible small-study effects; not definitive publication bias |
| Hypertension | 17 | -3.674 | 0.002 | Possible small-study effects; not definitive publication bias |
| Gross hematuria | 14 | -3.738 | 0.003 | Possible small-study effects; not definitive publication bias |
| Nephrotic-range proteinuria | 11 | -3.292 | 0.009 | Possible small-study effects; not definitive publication bias |
| RRT | 15 | -7.018 | <0.001 | Possible small-study effects; not definitive publication bias |
| Exploratory RPGN/crescentic-lesion composite | 10 | -6.729 | <0.001 | Possible small-study effects; not definitive publication bias |
| Study-reported severe complications | 16 | -3.801 | 0.002 | Possible small-study effects; not definitive publication bias |

Note: Funnel plots and Egger's regression were exploratory assessments of asymmetry and possible small-study effects. With only 10–18 studies per outcome and marked clinical/statistical heterogeneity, these methods have limited reliability for identifying publication bias; statistically significant asymmetry was therefore not interpreted as evidence that publication bias was present.

**Supplementary Table S7. Random-effects leave-one-out pooled-estimate ranges**

| **Outcome** | **No. leave-one-out analyses** | **Pooled range** | **Omission giving minimum** | **Omission giving maximum** | **I² range** |
| --- | --- | --- | --- | --- | --- |
| Study-defined AKI/acute kidney dysfunction | 18 | 37.8%-44.3% | Jellouli (2015) | Moges (2026) | 96.7%-97.8% |
| Hypertension | 17 | 63.2%-68.5% | Wan Yusof (2023) | Maalej (2018) | 93.5%-95.0% |
| Gross hematuria | 14 | 44.9%-52.5% | Curelaru (2025) | Moges (2026) | 96.1%-97.3% |
| Nephrotic-range proteinuria | 11 | 33.7%-38.7% | Hongsawong (2023) | Sepahi (2011) | 87.4%-90.6% |
| RRT | 15 | 2.9%-5.2% | El-Tigani (2014) | Wan Yusof (2023) | 86.6%-89.7% |
| Exploratory RPGN/crescentic-lesion composite | 10 | 8.4%-17.2% | Hongsawong (2023) | Maalej (2018) | 91.7%-95.7% |
| Study-reported severe complications | 16 | 11.6%-14.2% | Gebreyesus (2018) | Maalej (2018) | 78.2%-83.4% |

Note: Each analysis omitted one study while retaining the same random-effects framework used in the primary analysis.

**Supplementary Table S8. Adapted Newcastle-Ottawa Scale (NOS) quality assessment**

| **Study** | **S1** | **S2** | **S3** | **S4** | **Comparability (0-2)** | **O1** | **O2** | **O3** | **Total / quality** |
| --- | --- | --- | --- | --- | --- | --- | --- | --- | --- |
| Wong (2009) | — | ★ | ★ | — | ★ | ★ | ★ | — | 5/9 (Moderate) |
| Becquet (2010) | ★ | ★ | ★ | ★ | ★ | ★ | ★ | ★ | 8/9 (High) |
| Luo (2010) | — | ★ | ★ | — | ★ | ★ | ★ | ★ | 6/9 (Moderate) |
| Gunasekaran (2015) | ★ | ★ | ★ | ★ | ★ | ★ | ★ | ★ | 8/9 (High) |
| Jellouli (2015) | — | ★ | ★ | — | — | ★ | ★ | ★ | 5/9 (Moderate) |
| Ayoob (2016) | — | ★ | ★ | — | — | ★ | ★ | ★ | 5/9 (Moderate) |
| Demircioglu (2018) | ★ | ★ | ★ | ★ | ★ | ★ | ★ | ★ | 8/9 (High) |
| Gebreyesus (2018) | ★ | ★ | — | ★ | ★ | ★ | ★ | ★ | 7/9 (High) |
| Maalej (2018) | ★ | ★ | ★ | ★ | ★ | ★ | ★ | ★ | 8/9 (High) |
| Sepahi (2011) | — | ★ | — | ★ | ★ | ★ | ★ | ★ | 6/9 (Moderate) |
| Bajracharya (2024) | ★ | ★ | ★ | ★ | — | ★ | ★ | ★ | 7/9 (High) |
| Chong (2023) | ★ | ★ | ★ | ★ | ★ | ★ | ★ | ★ | 8/9 (High) |
| Wan Yusof (2023) | ★ | ★ | ★ | ★ | ★★ | ★ | ★ | ★ | 9/9 (High) |
| Moges (2026) | ★ | ★ | — | ★ | ★★ | ★ | ★ | ★ | 8/9 (High) |
| Curelaru (2025) | ★ | ★ | ★ | ★ | ★ | ★ | ★ | ★ | 8/9 (High) |
| Hongsawong (2023) | — | ★ | — | ★ | ★★ | ★ | ★ | ★ | 7/9 (High) |
| Dagan (2016) | — | ★ | — | ★ | ★★ | ★ | ★ | ★ | 7/9 (High) |
| El-Tigani (2014) | — | ★ | — | — | ★ | ★ | ★ | — | 4/9 (Low) |

NOS domain definitions: S1, representativeness; S2, clarity of cohort eligibility/source population; S3, APSGN ascertainment; S4, outcome-independent cohort selection; Comparability, up to 2 stars; O1, outcome ascertainment; O2, adequate observation window; O3, completeness of follow-up/reporting. High quality = 7-9 stars; moderate = 5-6; low = 0-4. The scale was adapted for predominantly single-cohort descriptive evidence.

**Supplementary Table S8A. Sensitivity analysis restricted to NOS high-quality studies (score >=7)**

| **Outcome** | **k** | **Pooled proportion** | **95% CI lower** | **95% CI upper** | **I² (%)** | **N** |
| --- | --- | --- | --- | --- | --- | --- |
| Study-defined AKI/acute kidney dysfunction | 12 | 34.6% | 22.0% | 54.4% | 97.1 | 1,879 |
| Hypertension | 12 | 66.8% | 51.1% | 87.4% | 95.8 | 1,897 |
| Gross hematuria | 10 | 43.6% | 27.1% | 69.9% | 98.0 | 1,467 |
| Nephrotic-range proteinuria | 7 | 38.3% | 26.5% | 55.4% | 90.3 | 679 |
| Renal replacement therapy | 10 | 1.4% | 0.6% | 3.0% | 58.8 | 1,231 |
| Exploratory RPGN/crescentic-lesion composite | 6 | 4.6% | 0.8% | 27.6% | 93.8 | 560 |
| Study-reported severe complications | 11 | 15.2% | 9.4% | 24.7% | 84.9 | 1,497 |

This quality-based sensitivity analysis used the same primary natural-log proportion, inverse-variance, DerSimonian-Laird, Hartung-Knapp random-effects framework as the main analysis using the final verified dataset. Jellouli (2015) scored 5/9 and was excluded from this high-quality subset and therefore did not contribute to these estimates.

**Supplementary Table S9. PROSPERO-prespecified ROBINS-I assessment for protocol transparency**

| **Study (year)** | **Bias due to confounding** | **Bias in selection of participants** | **Bias in classification of exposures/outcomes** | **Bias due to missing data** | **Bias in measurement of outcomes** | **Bias in selection of the reported result** | **Overall judgment** |
| --- | --- | --- | --- | --- | --- | --- | --- |
| Wong (2009) | Moderate | Moderate¹ | Low | Serious² | Low | Moderate³ | Serious |
| Becquet (2010) | Moderate | Moderate¹ | Low | Moderate | Low | Low | Moderate |
| Luo (2010) | Moderate | Moderate¹ | Low | Low | Low | Low | Moderate |
| Gunasekaran (2015) | Low | Low | Low | Low | Low | Low | Low |
| Jellouli (2015) | Moderate | Serious⁴ | Low | Moderate | Low | Low | Serious |
| Ayoob (2016) | Moderate | Serious⁴ | Low | Moderate | Low | Moderate | Serious |
| Demircioglu (2018) | Low | Moderate¹ | Low | Low | Low | Low | Moderate |
| Gebreyesus (2018) | Moderate | Moderate¹ | Low | Moderate | Low | Moderate³ | Moderate |
| Maalej (2018) | Moderate | Moderate¹ | Low | Serious² | Low | Low | Serious |
| Sepahi (2011) | Moderate | Moderate¹ | Low | Moderate | Low | Moderate³ | Moderate |
| Bajracharya (2024) | Moderate | Moderate¹ | Low | Moderate | Low | Low | Moderate |
| Chong (2023) | Moderate | Moderate¹ | Low | Serious² | Low | Moderate³ | Serious |
| Wan Yusof (2023) | Low | Moderate¹ | Low | Low | Low | Low | Moderate |
| Moges (2026) | Moderate | Moderate¹ | Low | Moderate | Low | Moderate³ | Moderate |
| Curelaru (2025) | Moderate | Moderate¹ | Low | Low | Low | Low | Moderate |
| Hongsawong (2023) | Low | Moderate¹ | Low | Moderate | Low | Low | Moderate |
| Dagan (2016) | Moderate | Moderate¹ | Low | Moderate | Low | Low | Moderate |
| El-Tigani (2014) | Moderate | Serious⁵ | Low | Serious² | Low | Moderate | Serious |

ROBINS-I was prespecified in PROSPERO. Because the instrument was developed for non-randomized intervention studies, the adapted NOS served as the primary quality-assessment instrument in this review; this table documents the prespecified approach for protocol transparency.

**Supplementary Figures**

**Supplementary Figure S1. Forest plot of the pooled proportion of gross hematuria across the included pediatric clinical cohorts.**

**Supplementary Figure S2. Forest plot of the pooled proportion of nephrotic-range proteinuria across the included pediatric clinical cohorts.**

**Supplementary Figure S3. Forest plot of the exploratory RPGN/crescentic-lesion composite across the included pediatric clinical cohorts. This heterogeneous composite is not a prevalence estimate for clinical RPGN or crescentic glomerulonephritis separately. Jellouli (2015) contributes 24/26 biopsied children. The primary-model prediction interval is capped at 100% for display; see the note to Table S5.**

**Supplementary Figure S4. Forest plot of the pooled proportion of study-reported severe complications across the included pediatric clinical cohorts.**

**Supplementary Figure S5. Random-effects leave-one-out sensitivity analysis for hypertension.**

**Supplementary Figure S6. Random-effects leave-one-out sensitivity analysis for study-defined AKI/acute kidney dysfunction.**

**Supplementary Figure S7. Random-effects leave-one-out sensitivity analysis for renal replacement therapy.**

**Supplementary Figure S8. Random-effects leave-one-out sensitivity analysis for gross hematuria.**

**Supplementary Figure S9. Random-effects leave-one-out sensitivity analysis for nephrotic-range proteinuria.**

**Supplementary Figure S10. Random-effects leave-one-out sensitivity analysis for the exploratory RPGN/crescentic-lesion composite. Prediction intervals are capped at 100% for display; see the note to Table S5.**

**Supplementary Figure S11. Random-effects leave-one-out sensitivity analysis for severe complications.**

**Supplementary Figure S12. Funnel plot for hypertension.**

**Supplementary Figure S13. Funnel plot for study-defined AKI/acute kidney dysfunction.**

**Supplementary Figure S14. Funnel plot for renal replacement therapy.**

**Supplementary Figure S15. Funnel plot for gross hematuria.**

**Supplementary Figure S16. Funnel plot for nephrotic-range proteinuria.**

**Supplementary Figure S17. Funnel plot for the exploratory RPGN/crescentic-lesion composite, using the Jellouli (2015) entry of 24/26 biopsied children.**

**Supplementary Figure S18. Funnel plot for severe complications.**
